# Mechanistic Diversity in Photoexcited Radical Redox Chemistry: A Critical Evaluation

**DOI:** 10.1002/anie.3889857

**Published:** 2026-08-11

**Authors:** Samuel J. Horsewill, Ryan Phelps, Joshua P. Barham, Daniel J. Scott

**Affiliations:** ^1^ Department of Chemistry University of Bath Bath UK; ^2^ Central Laser Facility Rutherford Appleton Laboratory Didcot UK; ^3^ Department of Pure & Applied Chemistry University of Strathclyde Glasgow UK

**Keywords:** conPET, e‐PRC, photoredox, radicals, transient absorption spectroscopy

## Abstract

“Photoexcited Radical Redox”, a subset of photoredox reactions in which electron transfer occurs via excitation of an already oxidized or reduced radical form of the photocatalyst, has been a cause of both great excitement and confusion in recent years. While the ability of photoexcited radical redox to access extreme redox potentials has led to a string of exciting, high‐profile, challenging new transformations, serious doubts have been raised as to the feasibility of the mechanisms proposed, leading to an extensive debate over a diversity of different mechanistic rationales. While these discussions are supported by a steadily growing body of experimental evidence, overall understanding of photoexcited radical redox remains murky at best. Each mechanistic model struggles to account for at least some important observations, and many important general questions remain unanswered. In this review, we comprehensively summarize the various mechanistic models that have been proposed to account for observations of (apparent) photoexcited radical redox, provide a detailed, critical assessment of the evidence for and against each of them, and highlight continuing gaps in collective understanding and directions for future investigations.

## Graphical abstract

Many high‐profile examples of photoredox catalysis are proposed to proceed via excitation of an open‐shell intermediate form of the catalyst. However, there is much debate over whether such mechanisms are realistic and, if so, exactly how they can occur. This review provides a comprehensive, critical summary of the current experimental evidence, its interpretations, and the limits of current understanding in this field.

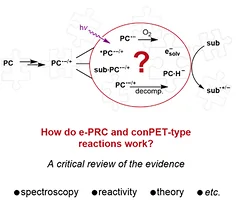

## 1 Introduction

Since the seminal modern reports of Yoon, Stephenson, and MacMillan in 2008/2009 [1, 2, 3], photoredox catalysis (PRC) has rapidly become one of the 21^st^ century's most important new synthetic methods [4]. The central concept of PRC is simple: photoexcitation of a photocatalyst (**PC**, almost always closed‐shell) into an excited state (*PC) grants it a higher energy electronic structure and hence notably enhanced redox power. This allows *PC to react with substrates via single electron transfer (SET) steps that would be energetically infeasible for the **PC** ground state. SET generates the corresponding one‐electron reduced or oxidized forms of **PC** (**PC** ^•−^ or **PC** ^•+^) that can then engage in a second SET step (which in ‘standard’ PRC is assumed to occur from/to the **PC** ^•−^/**PC** ^•+^ ground state) to close the catalytic cycle. Through this process, the energy of the original photon is used to transform one or more relatively unreactive substrates (**sub**) into much more reactive intermediates (**sub** ^•+^/**sub** ^•−^) that can then engage in further elementary reaction steps to yield the final products of interest (Scheme 1a). Note that depending on the charge on the initial **PC**, the **PC** ^•−^/**PC** ^•+^ state may not always be a formal anion/cation (e.g. if **PC** is a cation then **PC^•−^** will be neutral, as is the case for **Acr^+^**/**Acr** ^•^ shown in Figure 1).

**SCHEME 1:**
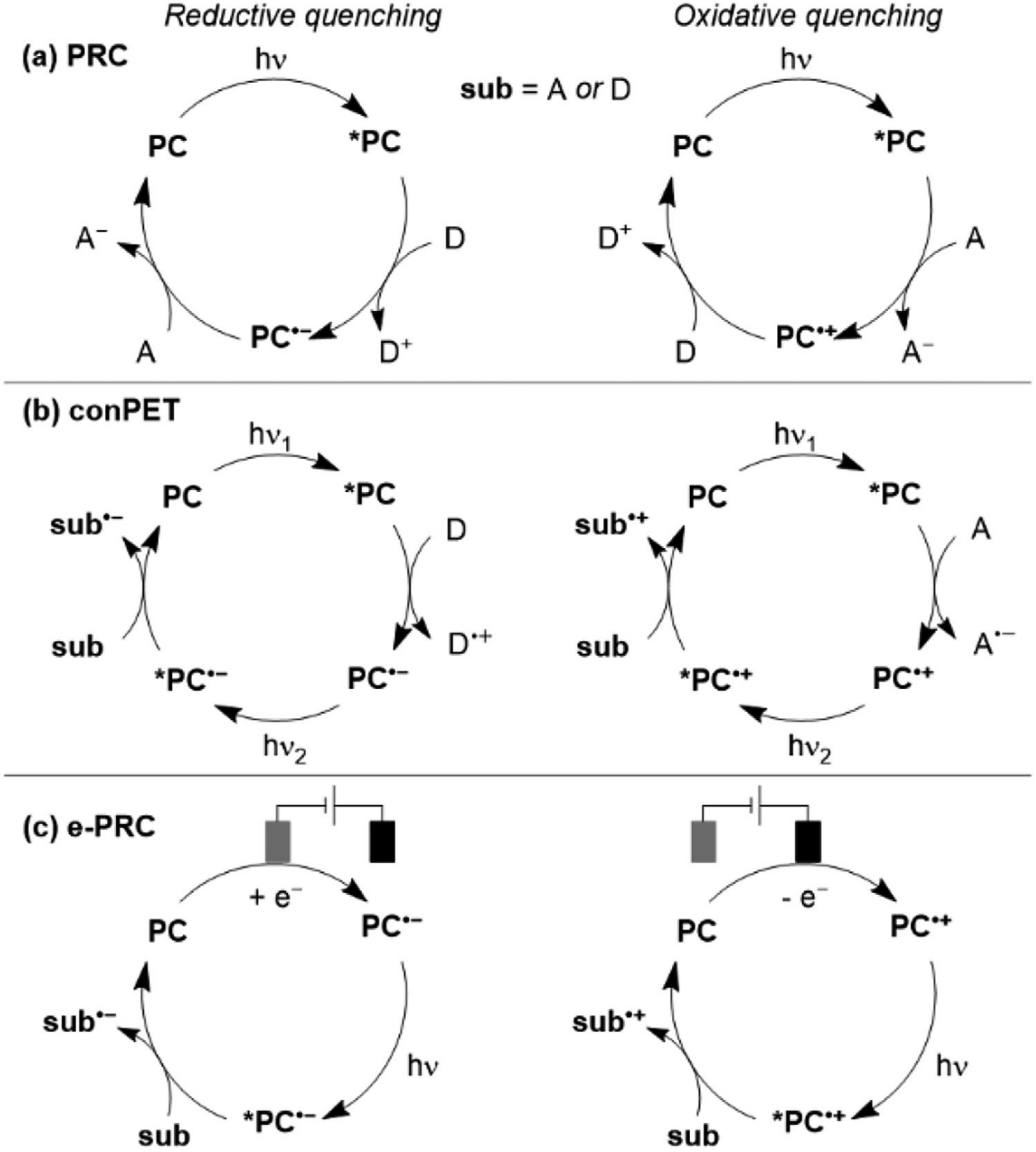
Generic mechanisms of (a) photoredox catalysis, (b) conPET, and (c) e‐PRC via reductive (left) or oxidative (right) quenching. **PC** = photocatalyst; **sub** = substrate; D = electron donor. A = electron acceptor.

**FIGURE 1:**
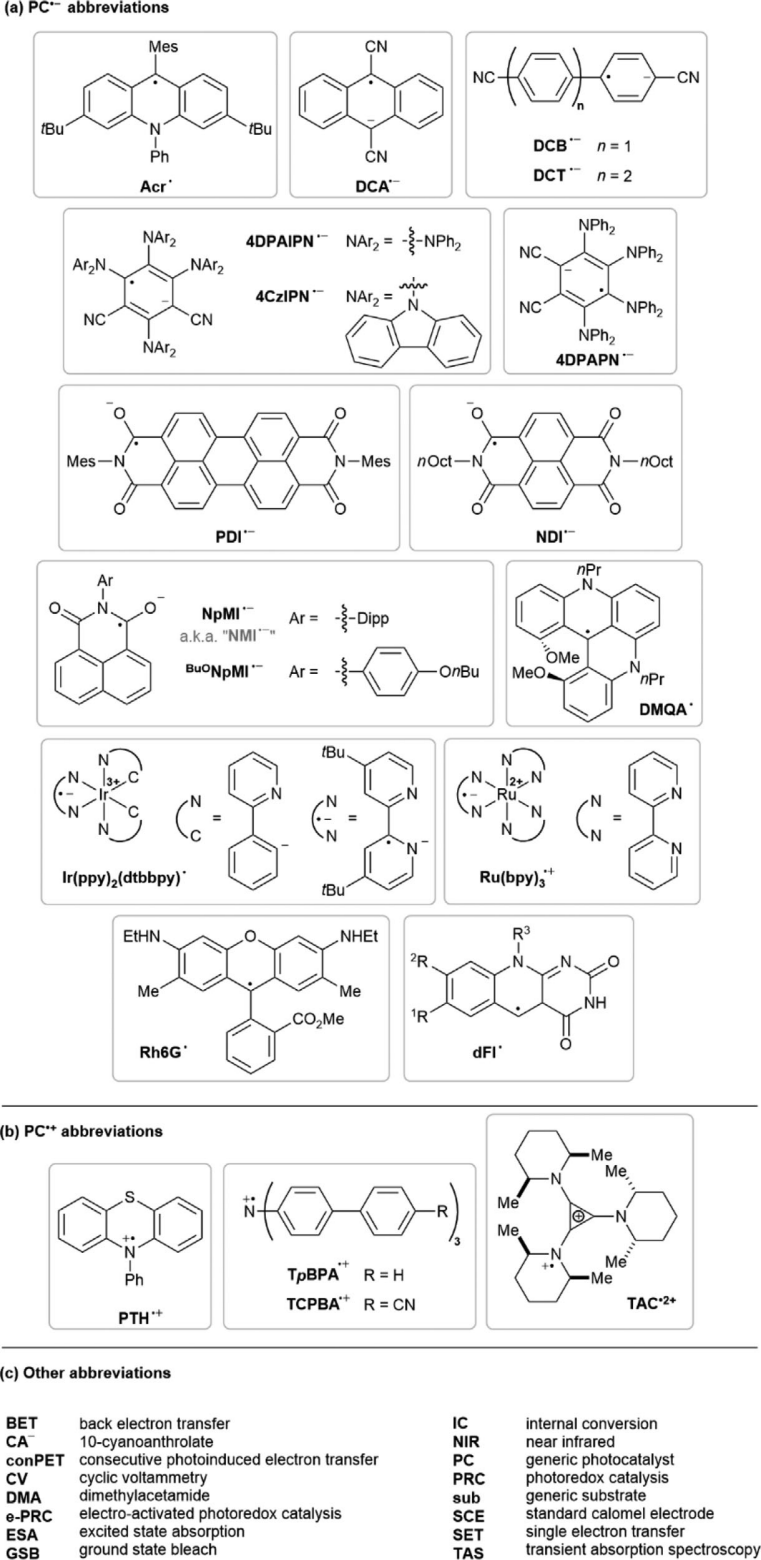
Glossary of key abbreviations for (a) **PC** ^•−^ states and (b) **PC** ^•+^ states, and (c) other non‐standard abbreviations. Dipp = 2,6‐di‐*iso*‐propylphenyl; Mes = 2,4,6‐trimethylphenyl.

The simplicity of this concept makes PRC extremely versatile, and many hundreds of PRC reactions have been reported using this operating platform [5, 6]. Nevertheless, PRC suffers from some well‐known limits, especially regarding the range of accessible redox potentials. Typically, to ensure selectivity, PRC reactions are driven by visible light (∼400–700 nm; sometimes also near UV, ∼350–400 nm) which will usually only be absorbed by the desired **PC**. Higher energy photons are avoided to minimize the risk of side‐reactions, which may be caused by undesired direct excitation of other components of the reaction mixture at these shorter wavelengths. Unfortunately, this inherently limits the energy, and hence redox potential, available in the *PC state, and thus the range of viable substrates, with effective redox limits of ±2 V vs. SCE being the most commonly quoted guideline [7]. This precludes functionalization of any particularly redox‐inert substrates (e.g. reduction of aryl chlorides or oxidation of simple arenes), many of which would be particularly attractive PRC feedstocks due to their stability and ready availability [8].

Various strategies have been proposed to overcome this limitation (e.g. via photon or redox upconversion, or bespoke design of more reducing/oxidizing *PC) [9, 10, 11, 12]. However, two of the most prominent and most successful are “consecutive photoinduced electron transfer” (conPET) and “electro‐activated photoredox catalysis” (e‐PRC) [11, 13, 14, 15, 16]. Although they differ synthetically, these two methods share a core mechanistic idea: rather than having the key SET step occur via excitation of the initial, closed‐shell **PC** state, it could instead occur from an already reduced/oxidized **PC** ^•−^/**PC** ^•+^. Thus, rather than photoexcitation being used only to transform a very weak redox agent (**PC**) into a strong one (*PC), it could instead be used to transform an already fairly powerful redox agent (**PC** ^•−^/**PC** ^•+^) into a very strong “super‐redox” agent (*PC ^•−^/*PC ^•+^) [13]. Indeed, relevant *PC ^•−^/*PC ^•+^ states have been reported to possess redox potentials that can match or even exceed those of elemental alkali metals (for *PC ^•−^ reductants; <−3 V vs. SCE) or the most potent oxidants such as F_2_ or Krossing's perfluorinated arenium cation ‘de‐electronators’ such as [C_10_F_8_]^•+^ (for *PC ^•+^ oxidants; >+3 V vs. SCE) [17, 18]. The **PC** ^•−^/**PC** ^•+^ themselves can be generated either photochemically, in direct analogy to ‘typical’ PRC (for conPET) or electrochemically (for e‐PRC) to give the net catalytic cycles summarized in Scheme 1b,c. (Note that the term ‘e‐PRC’ can also sometimes refer to ‘standard’ PRC mechanisms that include an electrochemical step, i.e. Scheme 1a where D or A is the working electrode; however, these are not relevant to this review [13, 14, 15].)

Since their initial descriptions in 2014 and 2019, respectively, conPET and e‐PRC have rapidly gained attention from the synthetic community, with dozens of often very high‐profile reports invoking these mechanisms to account for observed reactivity (for selected examples, see Scheme 2) [19, 20, 21, 22, 23, 24]. However, alongside this synthetic prominence has been substantial mechanistic controversy. Many researchers have questioned the feasibility of the key photoinduced SET steps proposed to occur from *PC ^•−^/*PC ^•+^ to substrate and have argued that alternative mechanisms must in fact be operative [25, 26]. However, this has been challenged by others [27], and this disagreement has led to a series of major mechanistic investigations that have resulted in a range of rationales, often divergent, each attempting to explain the photoreactivity observed experimentally [28]. These various mechanistic proposals, and the evidence for and against them, are the primary subject of this review.

**SCHEME 2:**
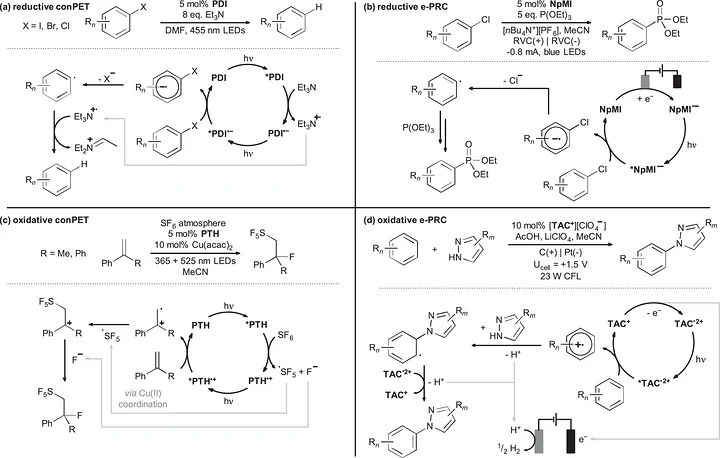
Seminal early synthetic examples of (a) reductive conPET, (b) reductive e‐PRC, (c) oxidative conPET, and (d) oxidative e‐PRC [19, 20, 22, 23], and mechanisms as originally proposed. For structures of the photocatalysts, see Figure 1. CFL = compact fluorescent lamp. RVC = reticulated vitreous carbon.

### 1.1 Review Scope

This review will focus specifically on the *mechanisms* of conPET, e‐PRC, and related reactions. Several comprehensive reviews of these methods’ synthetic applications are already available [11, 13, 14, 15, 16], and as such, this review will not focus on synthetic uses and is not intended to be an exhaustive survey of the literature regarding applications of these techniques. Instead, its aim is to provide a thorough, critical summary of the current, state‐of‐the‐art understanding of *how* these reactions occur, illustrated by selected, key examples. At the start of 2025, Pfund and Wenger provided an excellent Perspective on this field, which was also heavily mechanistically framed [28]. However, in the short time since then there have been several new milestone contributions. This review provides a more up‐to‐date summary, alongside more detailed and comprehensive analysis (to complement Pfund and Wenger's more focused approach). To ensure a holistic view of the field, this review has deliberately drawn upon the expertise of authors from distinct research backgrounds spanning organic synthesis (including applied photo‐ and electro‐synthetic methodologies and physical organic chemistry; JPB), inorganic chemistry (including isolation and study of reactive radicals; SJH, DJS), and ultrafast molecular spectroscopy (RP).

To the above ends, this review will begin by summarizing in turn each of the major mechanistic rationales that have been advanced as explanations of (apparent) conPET‐ or e‐PRC‐type reactivity. This will include the context in which each was originally proposed, the evidence that currently exists to support it, and any major experimental observations that the model cannot at present easily account for (Sections 2, 3, 4, 5). While this Review is primarily structured around individual mechanistic hypotheses, these Sections are also placed in loosely chronological order, to provide an indication of how ideas have evolved over the past 5–10 years. We hope that this will be particularly beneficial for newcomers to the field, including any synthetically focused practitioners who are interested in the potential of these methods but unfamiliar with the field's recent history. This will be followed by a more general discussion of the overall state of the literature (Section 6), with an emphasis on identifying important unanswered questions and the limits of current mechanistic understanding. This Section will highlight several important conclusions that can be drawn by considering the assembled literature in concert, including the current lack of evidence for a *single* universal mechanism, and the concomitant potential for multiple competing pathways to exist, varying on a case‐by‐case basis. Throughout this Review we have also chosen to discuss oxidative and reductive reactions in tandem rather than separately wherever possible, in order to emphasize the conceptual equivalence and mechanistic commonality of these two reaction sub‐classes.

#### 1.1.1 Guidance for Readers

This review aims to provide a *comprehensive* account of all significant evidence regarding the mechanisms of conPET/e‐PRC‐type reactions, alongside detailed, critical analysis. Sections 2, 3, 4, 5 each begin with a description of one major mechanistic rationale, followed by extensive discussion of the supporting evidence that has been reported. Section 6 then provides general discussion of the state of mechanistic understanding as a whole. Because this is a relatively long review, readers who are interested in this topic but do not require such comprehensive discussion are encouraged to focus their attention on the introductory portions of Sections 2, 3, 4, 5 and on Section 6, especially Section 6.5 which provides guidelines for future synthetically focused studies.

### 1.2 Notes on Terminology

Surprisingly, despite their recent prominence there is not yet a consistent, convenient shorthand term used to refer to SET reactions occurring to and from **PC** ^•−^/**PC** ^•+^ excited states. Although “conPET” and “e‐PRC” are well established, these terms carry additional synthetic and mechanistic implications (e.g. electrochemical generation of **PC** ^•−^/**PC** ^•+^ from **PC** for e‐PRC). Other possibilities tend to be vague and ambiguous (e.g. “radical photoredox”), clunky (e.g. “**PC** ^•−^/**PC** ^•+^ photoredox”), or overly narrow (e.g. “radical ion photoredox”, which would exclude **PC** ^•−^/**PC** ^•+^ states that lack formal net charge such as **PC** ^•−^ = **Acr^•^**, derived from **PC** = **Acr^+^**).

The abundance of radical intermediates throughout PRC makes it surprisingly difficult to provide a shorthand term that is entirely unambiguous (i.e. referring specifically to radicals in excited states, without also implying ground state radicals that were generated photochemically), and several options were considered while drafting this Review (e.g. “excited radical photoredox”). However, after discussion amongst ourselves as well as feedback during peer review, this Review has ultimately settled on the terms “photoexcited radical redox” and “photoexcited radical redox agents” to refer to these processes. We believe this captures the key, defining feature of these reactions (i.e. that specifically a *radical* species is photoexcited to act as a redox reagent) suitably precisely, while still remaining concise [29]. This term is intended to be ‘mechanistically agnostic’ and does not make any assumption as to whether the key SET step occurs via dynamic quenching (Section 2), static quenching (Section 4), or the intermediacy of solvated electrons (Section 5), requiring only that *PC ^•−^/*PC ^•+^ is actively involved in the redox process [30]. In cases where supposed photoexcited radical redox reactions may instead occur via a **PC** derivative other than *PC ^•−^/*PC ^•+^ (Section 3), this review will instead refer to *apparent* photoexcited radical redox (or similar).

Also important to this review is the possibility of photochemical reactions occurring from excited states other than the lowest excited state of a particular **PC** ^•−^/**PC** ^•+^, which in recent years has sometimes come to be referred to as “anti‐Kasha” reactivity [31], in reference to Kasha's rule [32]. This has become an increasingly common informal usage among synthetic chemists, and has also begun to find traction in the literature [9, 28, 31, 33]. However, while it provides a convenient shorthand, it should be emphasized that this usage remains highly contentious, especially outside the synthetically‐focused community, as Kasha's rule formally applies only to emission processes (and even in this context, the validity of “anti‐Kasha” terminology remains much debated) [34]. For the sake of caution, we will therefore avoid use of the term “anti‐Kasha” in this review, and instead refer to “higher doublet state” or “D*_n_* (*n* > 1)” reactivity [35].

It is also worth briefly noting here that, in contrast to ‘standard’ PRC where precious metal based photocatalysts remain the archetypes (Ru, Ir) [36], conPET and e‐PRC methods have from the start consistently tended towards the use of cheaper and more sustainable organic **PC** motifs [37]. As such, much of the mechanistic discussion below will implicitly assume an organic **PC** ^•−^/**PC** ^•+^ state (although most key arguments apply equally well to transition metal‐based **PC** ^•−^/**PC** ^•+^). Similarly, most detailed mechanistic studies to date have focused on *reductive* photoexcited radical redox processes (despite oxidative versions achieving at least as much, and arguably more, synthetic impact). Because of this, some discussions below will focus exclusively on **PC** ^•−^. However, in general the underlying arguments apply equally to **PC** ^•+^ (though *cf*. Section 5 for a notable exception).

## 2 Dynamic Quenching

### 2.1 The Problem with Dynamic Quenching

In the absence of any specific indication to the contrary, intermolecular, photoinduced SET is generally assumed to occur via dynamic quenching, i.e. via simple, diffusion limited encounters between **sub** and *PC, with a certain proportion of these encounters leading to productive SET.

As such, upon publication of the first synthetic reports of photoexcited radical redox, it was initially implicitly assumed that photoexcited radical redox events should also occur via dynamic quenching (Scheme 3a i) [19]. This supposition appeared tenable in early reports due to some descriptions of reasonably long‐lived **PC** ^•−^ excited state lifetimes (*τ_0_* = lifetime in the absence of substrate), [24, 38, 39] including a supposed lifetime of 13.5 ns for *DCA^•−^ and an *Acr^•^ lifetime apparently long enough to enable emissive behavior (though both were subsequently found to be mis‐attributions; *vide infra*). This is important, because excited state lifetimes play a critical role in determining the feasibility of photoinduced SET. In essence, the desired SET step is in competition with an undesired (and unproductive) relaxation of *PC ^•−^/*PC ^•+^ back to the **PC** ^•−^/**PC** ^•+^ ground state, which for organic motifs typically occurs via non‐radiative internal conversion (IC) processes (Scheme 3a ii). If this relaxation is rapid (i.e. if *τ_0_* is small), it is unlikely that the excited *PC ^•−^/*PC ^•+^ state will persist long enough and diffuse far enough to encounter a molecule of substrate, and the desired reaction will therefore become uncompetitive.

**SCHEME 3:**
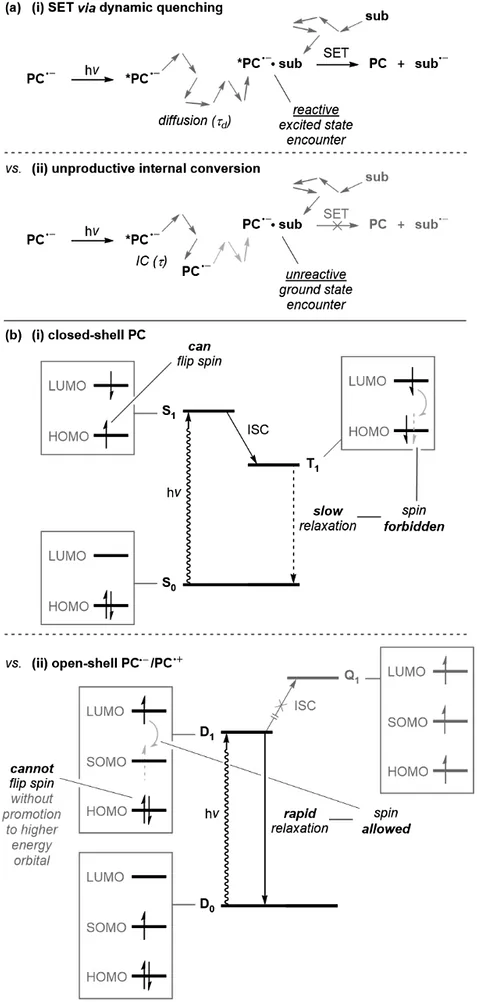
(a) Competition between (i) productive SET via dynamic quenching of a *PC ^•−^/*PC ^•+^ excited state and (ii) unproductive relaxation of the excited state via internal conversion. For simplicity, only the **PC** ^•−^ case is shown. (b) Jablonski and frontier MO illustration of (i) slow relaxation of triplet *PC vs. (ii) rapid relaxation of doublet *PC ^•−^/*PC ^•+^ (D_1_ is depicted as a SOMO‐to‐LUMO transition; depending on electronic structure, a HOMO‐to‐SOMO transition is also possible).

Under typical synthetic conditions, diffusion limited encounters between **PC** ^•−^/**PC** ^•+^ and substrate will generally occur with a lifetime (*τ*_d_) of approximately 1 ns between interactions, although the exact value will depend on factors such as concentration (*vide infra*) and solvent, since the diffusion lifetime for a given substrate concentration is closely related the diffusion‐limited reaction rate constant (*k*_d_), via:

(1) $$\tau_{d}=1/\left(k_{d}\left[\mathrm{sub}\right]\right)$$

The latter can vary appreciably between solvents (for convenience, some previously reported *k*_d_ values for a variety of solvents commonly used in PRC are given in Table 1; note that these calculations typically assume colliding particles to be neutral spheres of identical size) [40]. Nevertheless, a generally quoted ‘rule of thumb’ states that, based on typical diffusion lifetimes, excited state lifetimes of at least 1 ns are a prerequisite for efficient diffusion limited photoreactivity under typical synthetic reaction concentrations [37, 41, 42, 43].

**TABLE 1:** Diffusion limited rate constants, *k*_d_, at 298 K for a selection of common solvents^a^ [40].

Solvent	*k*_d_(M^−1^ s^−1^)
MeCN	1.9 × 10^10^
DMSO	3.3 × 10^9^
DMF	8.3 × 10^9^
DCM	1.6 × 10^10^
THF	1.4 × 10^10^
Et_2_O	3.0 × 10^10^
MeOH	1.2 × 10^10^
H_2_O	7.4 × 10^9^
PhMe	1.2 × 10^10^
hexane	2.2 × 10^10^

^a^ Values are generally calculated assuming colliding particles are neutral spheres of equal volume.

In more quantitative terms, the *maximum* quantum yield, *η*_max_, for a diffusion limited photoinduced SET step can be expressed as

(2) $$\eta_{max}=\tau_{0}/\left(\tau_{0}+\tau_{d}\right)$$

This represents an upper bound, because it assumes that *every* encounter between *PC ^•−^/*PC ^•+^ and substrate will result in a productive SET event (which in many cases may be very unrealistic, with most encounters leading to either no electronic change due to slow SET kinetics or to unproductive quenching/relaxation of the *PC ^•−^/*PC ^•+^ excited state). Assuming *τ*_d_ = 1 ns, this indicates that an excited state that matches the *τ*_0_ = 1 ns rule of thumb can potentially achieve synthetically useful SET quantum yields of up to 50% (i.e. productive SET and unproductive IC occurring at essentially equal rates). This increases to ca. 90% for an excited state lifetime of 10 ns. In contrast, sub‐ns lifetimes of 100, 10, or 1 ps imply much lower maximum efficiencies of ca. 10%, 1%, or just 0.1% respectively. Given that they are upper bounds, these are likely to be inconsistent with *efficient* photoinduced reactivity, although it should be emphasized that even *inefficient* photochemical steps may lead to substantial substrate conversion upon sufficiently prolonged irradiation. (Indeed, many other photoredox processes are known to require extended reaction times to compensate for low net quantum yields, often <1%. Unfortunately, quantum yields have generally not been quantified during studies of photoexcited radical redox, and are under‐reported for PRC as a whole [44].)

Unfortunately, the *actual* lifetimes of relevant *PC ^•−^/*PC ^•+^ states are typically extremely short, often as low as just a few ps (e.g. *NpMI^•−^, 24 ps [26]; *4CzIPN^•−^, 27 ps [45]; *T*p*BPA^•+^, 5 ps [46]). Moreover, some of the longer lifetimes that had been invoked in early synthetic reports were noted to be erroneous. For example, the 13.5 ns lifetime of *DCA^•−^ quoted in the original synthetic report of **DCA**'s use for e‐PRC was taken from a 1983 paper in which this value was obtained via emission quenching experiments [47]. However, subsequent work had shown this emission was misassigned. In fact, it originated from an impurity, 10‐cyanoanthrolate (**CA^−^**; *vide infra*, Section 3.1), generated due to incomplete exclusion of O_2_ during in situ generation of **DCA^•−^** (Scheme 4) [33, 48]. Subsequent studies have shown the *DCA^•−^ lifetime to be much shorter [49, 50], with the most recent works providing a value of just ∼3 ps [33, 51]. The much shorter excited state lifetimes of these open‐shell **PC** ^•−^/**PC** ^•+^ states compared to the corresponding open‐shell **PC**, and especially **PC** *triplet* states (e.g. *4CzIPN, 13.4 ns [45]; *DCA, 14.9 ns [52]), can be fairly easily rationalized based on differences in spin multiplicity. For the latter, direct triplet relaxation to singlet ground states is spin forbidden (*cf*. spin‐allowed relaxation of doublet excited states to doublet ground states; Scheme 3b). Note, however, that relevant **PC** *singlet* excited states can also be relatively long‐lived (>1 ns).

**SCHEME 4:**
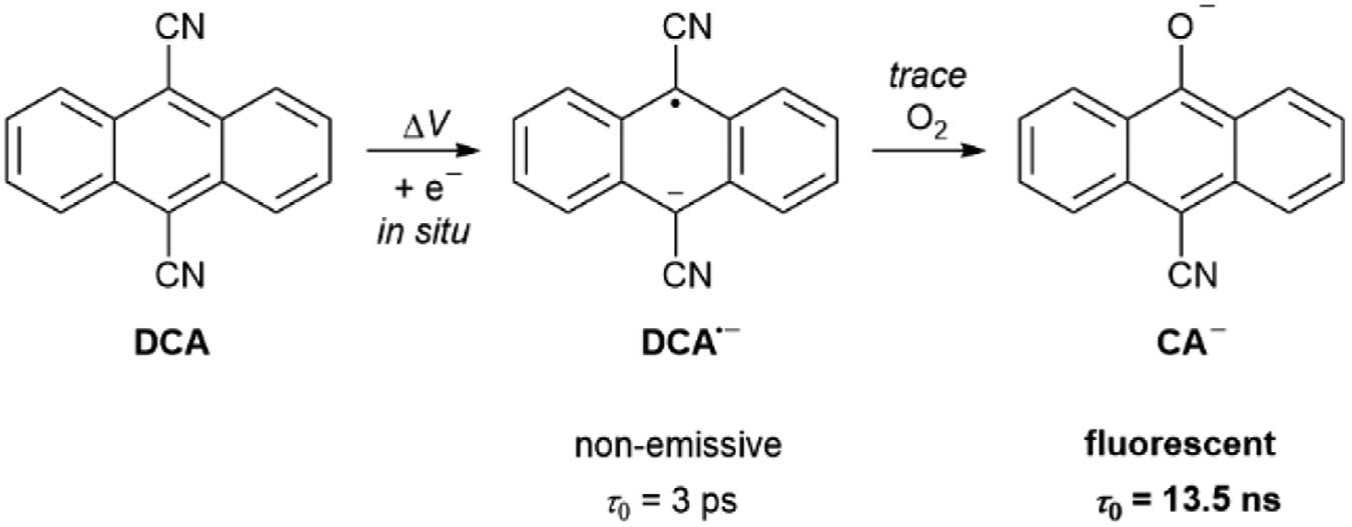
Mistaken attribution of a relatively long (ns) excited state lifetime to *DCA^•−^ caused by in situ decomposition into **CA^−^**.

One of the first reports to clearly highlight this lifetime problem came in 2018 from Cozzi, Ceroni, and colleagues [25], who investigated the mechanism proposed in the original report of synthetic conPET by König and coworkers in 2014 [19], which had used **PDI** as **PC** (*cf*. Scheme 2a). This investigation heavily questioned the feasibility of the proposed photoexcited radical redox step, based on the short (sub‐ns) *PDI^•−^ excited state lifetime of just 145 ps (**PDI^•−^** also absorbs strongly only above ∼600 nm, and barely at all at the shorter wavelengths used synthetically: 455 nm LEDs). Instead, based on in situ spectroscopic monitoring, it was proposed that the observed photoreactivity must instead be due to degradation of **PDI^•−^** and conversion into an alternative photoreductant. However, attempts to identify this key species were inconclusive (and subsequent studies have implied that dynamic quenching *can* in fact be possible; see Section 2.2).

### 2.2 Exceptions to the Lifetime Problem (for Dynamic Quenching)

The lifetime arguments against dynamic quenching of *PC ^•−^/*PC ^•+^ states are strong. However, it should be noted that there are, in principle, three ways in which this lifetime problem can be overcome:

- by increasing the relevant *PC ^•−^/*PC ^•+^ lifetime;
- by increasing the substrate concentration beyond ‘typical’ levels used in synthesis (e.g. significantly over 100 mM); or
- by accepting a very poor reaction quantum yield (and hence poor energy efficiency) and compensating through use of extended irradiation times (provided the photocatalytic system is sufficiently photostable, though in practice **PC** motifs are frequently prone to decomposition; *cf*. Section 3).

In fact, despite the concerns around **PDI^•−^** described in the previous Section, Zhang, Schanze, and colleagues have more recently reported that *PDI^•−^ (generated in situ via chemical reduction with TDAE in DMF; Scheme 5) *can* in fact engage in productive dynamic quenching under some conditions (700 nm excitation, consistent with the **PDI^•−^** absorption spectrum), albeit using relatively high substrate concentrations (hundreds of mM) and only for certain substrates (quenching was not observed for more challenging, harder to reduce substrates, despite these being employed successfully in the original synthetic study; it was suggested that this may indicate divergent mechanisms) [27]. In this study, ultrafast transient absorption spectroscopy (TAS) [43] measurements confirmed quenching of the *PDI^•−^ lifetime (for example, from 160 ps in the absence of substrate to 87 ps in the presence of 400 mM 4‐nitrobenzaldehyde, **1**; −0.86 V reduction potential vs. SCE, *cf*. −0.43 V for the ground state **PDI**/**PDI** ^•−^ couple), with Stern‐Volmer analysis providing dynamic quenching rate constants that approach the diffusion limit for the easiest‐to‐reduce substrates (∼2 × 10^10^ M^−1^ s^−1^). TAS experiments for these easiest substrates further confirmed SET from *PDI^•−^ through observation of persistent (> 1 ns) new absorptions and ground state bleach (GSB) features consistent with formation of **PDI** and loss of **PDI^•−^**, respectively (Scheme 5). As well as photoinduced SET, for some aryl halide substrates the authors also observed a slower, ground state SET reaction. Although the initial SET steps should be very uphill (e.g. by >1.2 V for 4‐iodobenzaldehyde), it was proposed that the overall reactions are facilitated by extremely rapid (and irreversible) subsequent C–X bond cleavage.

**SCHEME 5:**
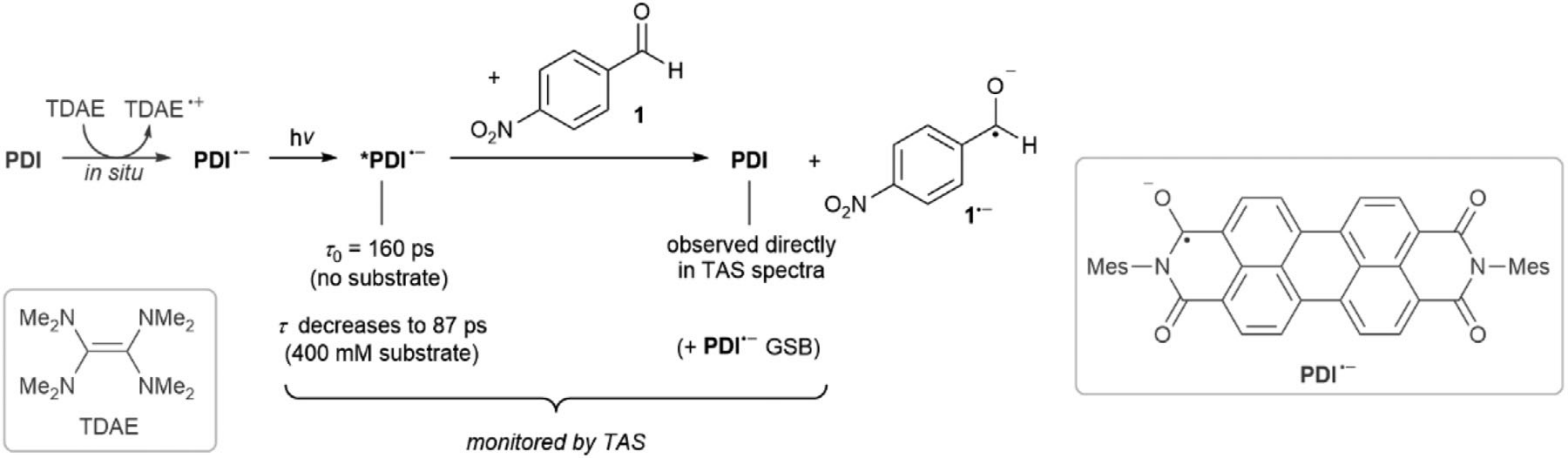
Observation of dynamic quenching of *PDI^•−^ by electron‐accepting substrates via TAS measurements.

Very recently, the feasibility of dynamic quenching for **PDI^•−^** has been further supported by the Fantin group, who investigated the electrochemical behavior of **PDI** by cyclic voltammetry (CV) [53]. This study found no difference between the **PDI** → **PDI^•−^** reduction steps in the presence or absence of organohalide substrates in the dark, consistent with the expected lack of ground state SET. However, under relatively long wavelength irradiation (630 nm; *cf*. 455 nm in the original synthetic report [19]), designed to selectively excite **PDI^•−^**, an enhanced current was observed, but only in the presence of relatively easy to reduce substrates. This is consistent with the anticipated photoinduced SET from *PDI^•−^ to substrate. Quantitative modelling of the electrochemical data indicated that this SET occurs at rates consistent with diffusion, but is extremely inefficient, with only ∼0.1% quantum yield for the desired reaction per **PDI^•−^** photoexcitation. The authors also noted that a lack of any CV response to the presence of even large excesses of substrate in the dark suggests a lack of any significant pre‐association between **PDI^•−^** and substrate (*cf*. Section 4), as even weak interactions would be expected to cause anodic shifts (i.e. shifts to more positive potential). This electrochemical strategy is potentially an elegant complement to the more commonly employed TAS investigations of *PC ^•−^/*PC ^•+^. However, it should be emphasized that this method was only able to provide accurate estimates of SET rate constants for relatively easy to reduce substrates, with reduction potentials only slightly higher than the *ground state* **PDI**/**PDI** ^•−^ couple (−0.52 to −0.46 V and −0.41 V, respectively, all vs. SCE), and not for the more challenging substrates typically of interest in catalytic contexts. In principle, there may also be differences between redox behavior occurring at the electrode surface (as captured by CV) and in bulk solution (where the majority of **PC** ^•−^/**PC** ^•+^ excitation is likely to occur during synthetic procedures).

The successful dynamic quenching of **PDI^•−^** can be attributed to a combination of all three of the factors noted above: i.e. a *PC ^•−^ lifetime that, while <1 ns, is substantially higher (>100 ps) than for most other **PC** ^•−^; relatively high substrate concentrations that decrease *τ*_d_ sufficiently below 1 ns to enable SET to become competitive; and a quantum yield that is nevertheless very low. More recently, the Schanze group have reported a very similar study on a closely related radical anion, **NDI^•−^**. Again, dynamic quenching was reported to be possible due to a directly analogous combination of high substrate concentration and relatively high *NDI^•−^ lifetime (200 ps) [54].

More broadly, it can be noted that although *most* open‐shell organic compounds display extremely short excited state lifetimes (including, to our knowledge, sub‐ns lifetimes for *all* relevant examples of **PC** ^•−^/**PC** ^•+^ states), this need not automatically be the case. Through careful molecular design, many stable, emissive radicals have been designed with excited state lifetimes well over 1 ns [55, 56, 57], although to date these have not to our knowledge been investigated in conPET or e‐PRC applications. Nevertheless, great care should be taken when assigning a long‐excited state lifetime to a **PC** ^•−^ or **PC** ^•+^ state, as *apparent* evidence of such long lifetimes can easily be misattributed, as already discussed in the case of **DCA^•−^** vs. **CA^−^** (Scheme 4). More recently, we (SJH, DJS) have shown that a long‐lived (ca. 10 ns) emission that had previously been assigned to **Acr** ^•^ [38, 39], and which it had subsequently been suggested might be attributable to a similar extrinsic sample contamination [28], is in fact an emission from closed‐shell **Acr^+^**, which is intrinsically generated in situ following initial photoexcitation (Scheme 6; see also Section 5.2) [58].

**SCHEME 6:**
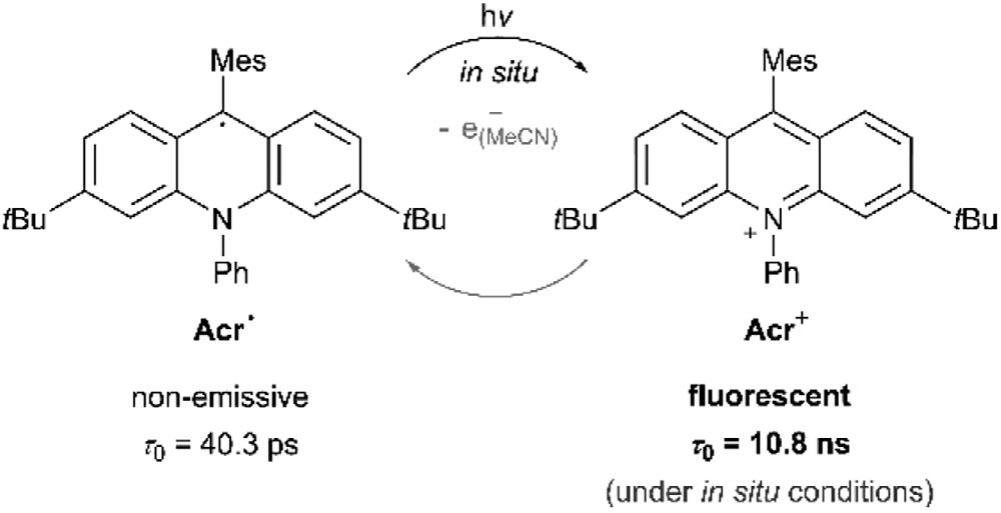
Apparent long‐lived fluorescence of **Acr^•^** caused by in situ formation of **Acr^+^**.

Whereas (re‐)designing a photocatalyst to achieve a long‐lived *PC ^•−^/*PC ^•+^ state is far from simple, increasing substrate concentration is often very straightforward, especially for substrates that are relatively inexpensive and can therefore comfortably be used in excess [59]. This strategy may therefore deserve more frequent consideration in future. Indeed, high substrate concentration has been a feature of some other prominent examples of apparently successful photoexcited radical redox. For example, in one of the early examples of oxidative e‐PRC, we (JPB, DJS) described the oxidation of even very challenging arenes (e.g. PhCl) using biaryl‐based triarylaminium photooxidants such as **T*p*BPA^•+^** and **TCPBA^•+^**, with the arenes being used in concentrations exceeding 300 mM in certain cases [46]. In this case, experimental and DFT evidence indicated a *structured* pre‐assembly (supported by subsequent molecular dynamics and TAS studies; *vide infra*, Scheme 12) [60, 61]. However, it should be noted that at sufficiently high substrate concentrations it also becomes increasingly likely that on a purely statistical basis, one or more substrate molecules will be found in the solvation shell of **PC** ^•−^/**PC** ^•+^ prior to photoexcitation. In this scenario, diffusion limited dynamic quenching will begin to give way to *static* quenching (see Section 4). For example, the Wickens group have reported oxidative arene functionalization reactions (similar to those shown in Scheme 2b) catalyzed by **PTH** via a proposed photoexcited radical redox mechanism (conPET) in which the arene substrate provides ∼50% of the total reaction volume (>5 M) [62].

## 3 Catalysis via PC^•−^/PC^•+^ Evolution into Closed‐Shell Photoredox Agents

Despite the specific exceptions discussed in Section 2.2, in general the lifetime argument against *efficient* dynamic quenching of *PC ^•−^/*PC ^•+^ states is robust. This was problematic for early conPET/e‐PRC mechanistic proposals, suggesting that other mechanisms might actually be operative (especially for examples where particularly high substrate concentrations were *not* reported) likely via in situ conversion of **PC** ^•−^/**PC** ^•+^ into alternative, *closed‐shell*, photoredox‐active intermediates (**PC** ^deg^, Scheme 7).

**SCHEME 7:**
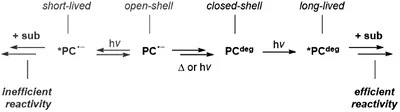
Possible *apparent* photoexcited radical reactivity via decomposition of **PC** ^•−^/**PC** ^•+^ into a degradation product, **PC** ^deg^ in situ. For simplicity, only the **PC** ^•−^ case is shown.

### 3.1 PC^•−^ Over‐Reduction or Oxidation

Alongside Cozzi and Ceroni's 2018 report discussed in Section 2.1, one of the most prominent early challenges to the idea of photoexcited radical redox agents was presented in 2021 by Nocera and coworkers [26]. Based on very similar lifetime principles, these authors argued that the reductive e‐PRC mechanisms originally proposed for **NpMI** were infeasible, and this view was supported by measurement of a very short *NpMI^•−^ lifetime of just 24 ps, which is almost an order of magnitude lower than the (already problematic) lifetime of *PDI^•−^ (Section 2.1). As an alternative photoreductant to **NpMI^•−^** the authors identified a closed‐shell, dearomatised structure (described as “Meisenheimer‐type”), **NpMI•H^−^** (Scheme 8a). Formally a hydride adduct of **NpMI**, **NpMI•H^−^** is better thought of as the result of over‐reduction of **NpMI^•−^** to **NpMI^2−^**, coupled with protonation of this presumably very strongly basic (and/or photobasic) dianion.

**SCHEME 8:**
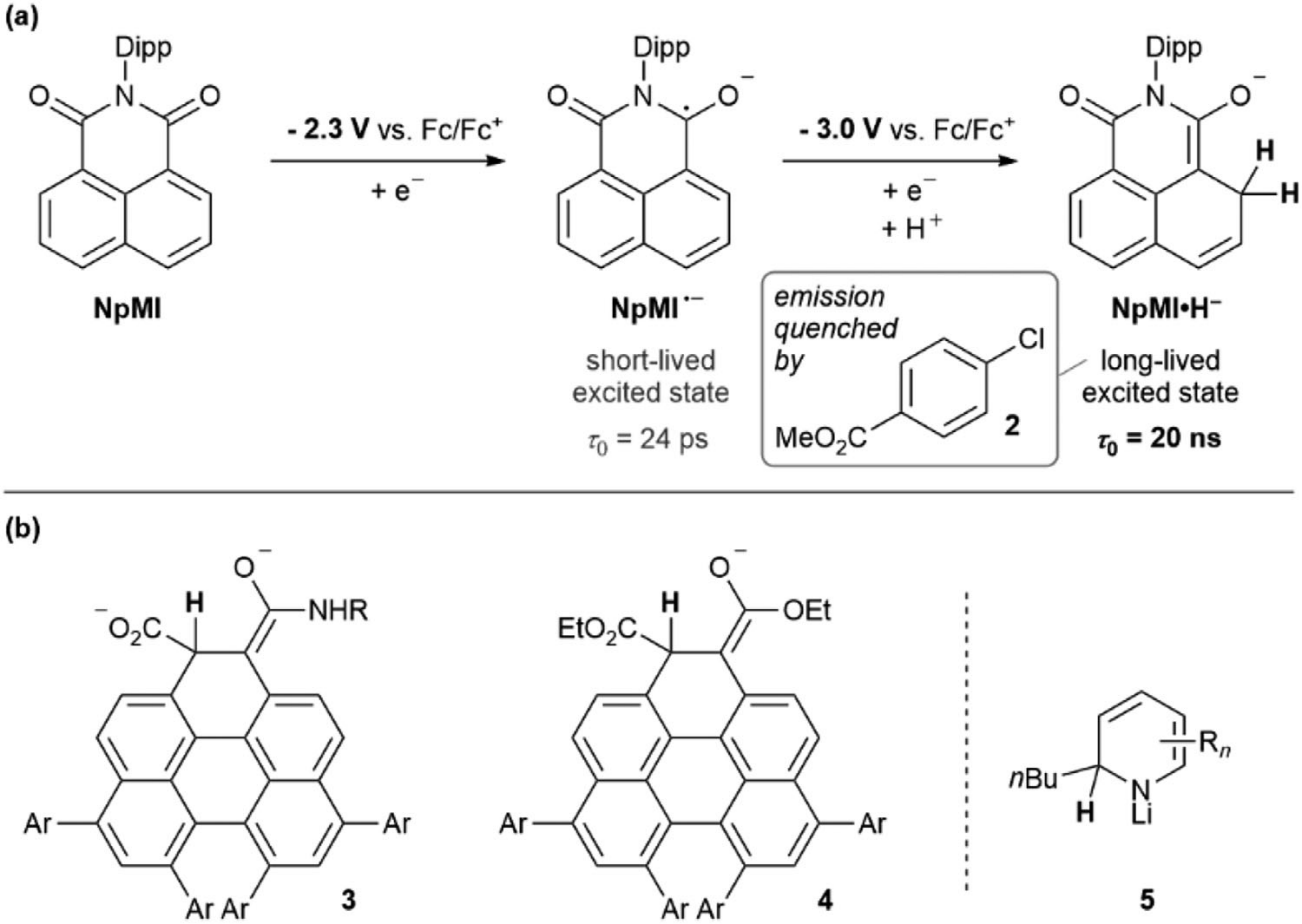
(a) Conversion of **NpMI^•−^** into **NpMI•H^−^** as an alternative in situ photoreductant, and (b) analogous **PC**•H^−^‐type intermediates subsequently invoked in strongly reducing photocatalysis.

Crucially, it was shown that **NpMI•H^−^** can be generated via bulk electrolysis of **NpMI** in dimethylacetamide (DMA) solution at a very negative potential: −3.0 V vs. Fc/Fc^+^ (ca. −2.6 V vs. SCE) [63]. For comparison, electrolysis at −2.3 V vs. Fc/Fc^+^ (ca. −1.9 V vs. SCE) was found to generate **NpMI^•−^** only. The identity of **NpMI•H^−^** was corroborated by UV‐vis comparison with isolated samples of [*n*Bu_4_N^+^][**NpMI•H^−^**] prepared chemically (via NaBH_4_ reduction of **NpMI**), whose structure was authenticated by NMR and XRD analysis. Moreover, it was shown that photoexcitation of **NpMI•H^−^**, which absorbs broadly in the 400‐550 nm region, leads to a long‐lived, emissive excited state (*τ*_0_ = 19.7 ns) well suited for dynamic quenching. Indeed, this emission lifetime was indeed decreased by the presence of a model substrate, methyl 4‐chlorobenzoate (**2**), in a manner consistent with dynamic quenching. The authors also described quenching by PhCl, but in this case the observed effect was minimal, with the lifetime decreasing only to 18.2 ns (1.5 ns change, ∼7%) even with 400 mM PhCl (*vide infra*).

As a result, it was proposed that the *apparent* photoexcited radical redox reactivity observed for **NpMI^•−^** is in fact the result of in situ over‐reduction of **NpMI^•−^** to **NpMI•H^−^**, which is then a much more viable photoreductant. By extension, it was also suggested that apparent photoexcited radical redox processes using other **PC** ^•−^ are likely due to analogous in situ changes. Indeed, Miyake, Damrauer, and colleagues have subsequently shown evidence for similar **PC**•H^−^ intermediates (**3**, **4**) in photoreduction chemistry using **PC** structures based on more extended polyaromatic frameworks (Scheme 8b, left and middle) and have exploited the deep photoreducing power of these species to design a series of high impact transformations [64, 65, 66, 67, 68]. Similarly, a team led by Negri, Ceroni, and Cozzi have achieved strongly reducing photocatalysis by deliberately targeting in situ generation of stoichiometric Meisenheimer‐type adducts from pyridines and *n*BuLi (**5**, Scheme 8b, right) [69, 70].

In another report, the Nocera group have more recently applied a similar logic to conPET reactions of **DCA^•−^** [33]. As already noted, **DCA^•−^** has a very short excited state lifetime (3 ps) but exposure to O_2_ leads to facile conversion into **CA^−^** (Scheme 4). Given the much longer lifetime of *CA^−^ (13.5 ns) and the fact that related aryloxides (i.e. ArO^−^) have independently been shown to be powerful photoreductants [71, 72, 73], several authors [13, 28, 74] have noted the possibility that **CA^−^** could be playing a major, productive role in the originally observed synthetic transformations. This was validated by the Nocera group by showing that photoexcitation of authentically isolated K^+^[**CA^−^**] in the presence of ethyl 4‐chlorobenzoate does indeed lead to quenching of emission from the former (in spectroscopic studies), as well as reductive functionalization of the latter (in reactivity studies using stoichiometric K^+^[**CA^−^**]), albeit only in quite low yield (22%, Scheme 9) [33]. No quenching or productive reduction was observed for the more challenging substrates PhCl or 4‐chloroanisole (**6**), however (*vide infra*, e.g. Figure 2).

**SCHEME 9:**
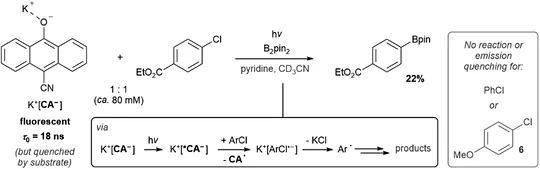
Photoreaction between ethyl 4‐chlorobenzoate, **6**, and **CA^−^** (which can be formed by exposure of **DCA^•−^** to O_2_).

**FIGURE 2:**
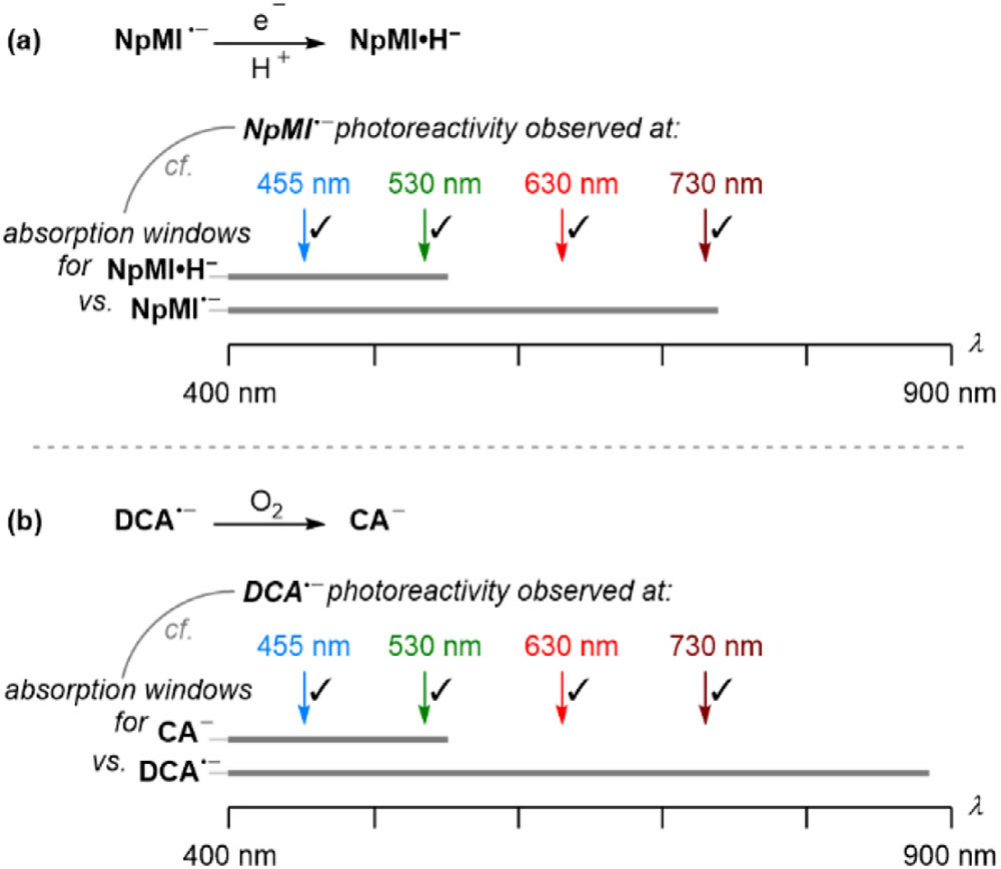
UV‐vis absorption windows for (a) **NpMI^•−^** vs. **NpMI•H^−^**; and (b) **DCA^•−^** vs. **CA^−^** and comparison with observed photoreactivity of isolated **PC** ^•−^.

In one sense, the evidence presented by these reports is unambiguous: clearly, **PC** ^•−^ *can* evolve into strong, closed‐shell photoreductants, and these new species *can* induce reduction of synthetically relevant substrates upon irradiation, at least for certain **PC** ^•−^. The remaining questions, then, are whether these species actually do form under synthetically relevant conditions and, if so, whether their reactivity profiles match the synthetic results; and whether they are capable of catalytic turnover under conditions relevant to actual photoexcited radical redox reactions.

Here the evidence is much less conclusive. For example, while the reported electrochemical generation of **NpMI•H^−^** is *qualitatively* consistent with the cathodic generation of **NpMI^•−^** from **NpMI** (*E*_1/2_ = −1.3 vs. SCE [23]) during synthetic e‐PRC, it is notable that the potential used to do so (−3.0 V vs. Fc/Fc^+^ ≈ −2.6 V vs. SCE [63]) is substantially more negative that the potential applied synthetically (e.g. −1.3 V vs. SCE for hydrodehalogenation of 4‐bromobiphenyl, coinciding with the **NpMI**/**NpMI** ^•−^ couple [23]), implying that **NpMI•H^−^** may not necessarily be accessible. In addition, UV‐vis analysis of **NpMI•H^−^** shows significant absorption bands only at wavelengths below ca. 550 nm. However, as will be discussed in the next Section, studies using authentically isolated **NpMI^•−^** have shown apparent photoexcited radical redox occurring under 630 or 730 nm irradiation, which should not be capable of exciting any theoretically in situ generated **NpMI•H^−^** (Figure 2a). Excitation of **NpMI^•−^** has also been shown to induce reduction of challenging substrates (<−2 V vs. SCE) with which **NpMI•H^−^** appears to struggle, such as PhCl and even 4‐chloroanisole, **6** (*cf*. Scheme 8a) [74]. It is also worth noting that, whereas synthetic e‐PRC reactions using **NpMI** have generally been performed in MeCN or DMSO, the study of **NpMI•H^−^** was carried out in DMA. It is reasonable to assume that this difference will not dramatically impact either the redox behaviour of **NpMI^•−^** or the photochemical behaviour of **NpMI•H^−^**. However, as the complexity of conPET and e‐PRC mechanisms has become steadily more evident, it has become increasingly desirable for mechanistic studies to match the conditions of actual synthesis as closely as possible (see Section 6.3).

Similar observations can be made regarding **CA^−^**. Given the established transformation of **DCA^•−^** into **CA^−^** upon exposure to O_2_, the formation of **CA^−^** during synthetic conPET or e‐PRC reactions is certainly plausible [75]. Studies on isolated **PC** ^•−^ have consistently confirmed their extreme air‐sensitivity, mandating use of rigorous air exclusion techniques (i.e. Schlenk line, glovebox) to avoid decomposition. While reductive conPET and e‐PRC syntheses do tend to be performed under nominally inert atmospheres, in practice this is usually limited to more accessible but less rigorous methods such as N_2_/Ar sparging and balloons (indeed, the need for more laborious air‐sensitive methods would be a major practical drawback). As such, minor O_2_ exposure (at least) is very likely. For example, in e‐PRC, electrodes must often pierce through septa, inevitably providing gaps for air ingress. Nevertheless, this can again be contrasted with experiments using isolated **DCA^•−^** (see Section 4.1) performed under much stricter O_2_‐free conditions [74]. As with isolated **NpMI^•−^**, these showed clear evidence of apparent photoexcited radical redox reactivity, despite the absence of any likely pathway for **CA^−^** formation, as well as reactions occurring under 630 or 730 nm irradiation for certain substrates, despite **CA^−^** not absorbing above ca. 550 nm (Figure 2b). Irradiation of authentic **DCA^•−^** at shorter wavelengths also enabled reduction of challenging substrates such as PhCl and 4‐chloroanisole, **6** (which were also successfully functionalized in synthetic studies using **DCA**) [24], despite these seemingly being unreactive towards **CA** ^−^ (*cf*. Scheme 9) [33]. Moreover, CV analysis of **CA^−^** suggests that the **CA** ^•^ radical formed after photoinduced SET is a very weak oxidant (0.07 V vs. SCE) [33], which is seemingly inconsistent with ground‐state regeneration of **CA^−^** (as required for catalytic turnover) for some of the terminal oxidants that have been employed synthetically (e.g. tertiary amines, ∼1 V vs. SCE).

### 3.2 Other Pathways

Though few other examples have been studied in equivalent detail, the range of pathways by which **PC** ^•−^ might be degraded into alternative, closed‐shell photoreductants is clearly not limited to formation of “Meisenheimer‐type” **PC**•H^−^ adducts and oxidation of **DCA^•−^** to **CA^−^** by O_2_. For example, in their initial critical study of **PDI^•−^** (discussed in Section 2.1) Cozzi, Ceroni, and co‐workers noted that its irradiation in the presence of 4’‐bromoacetophenone led to clear decomposition (although the products of this decomposition were not identified) [25]. Meanwhile, we have shown that **DCA^•−^** appears to be unstable in the presence of aryl radicals formed during ArCl reduction reactions, which we suggested may be due to radical addition to the C–CN sites and loss of CN^−^ (forming **7**, Scheme 10a) [74]. This is supported by similar observations from the König group (and others), who have described analogous substitution of CN via radical attack on **4CzIPN^•−^** (forming **4CzBnBN**, Scheme 10b) [76, 77]. While this latter study was not specifically focused on photoexcited radical redox, **4CzIPN** and related structures have since attracted significant interest in conPET/e‐PRC applications (see Section 5.1) [78].

**SCHEME 10:**
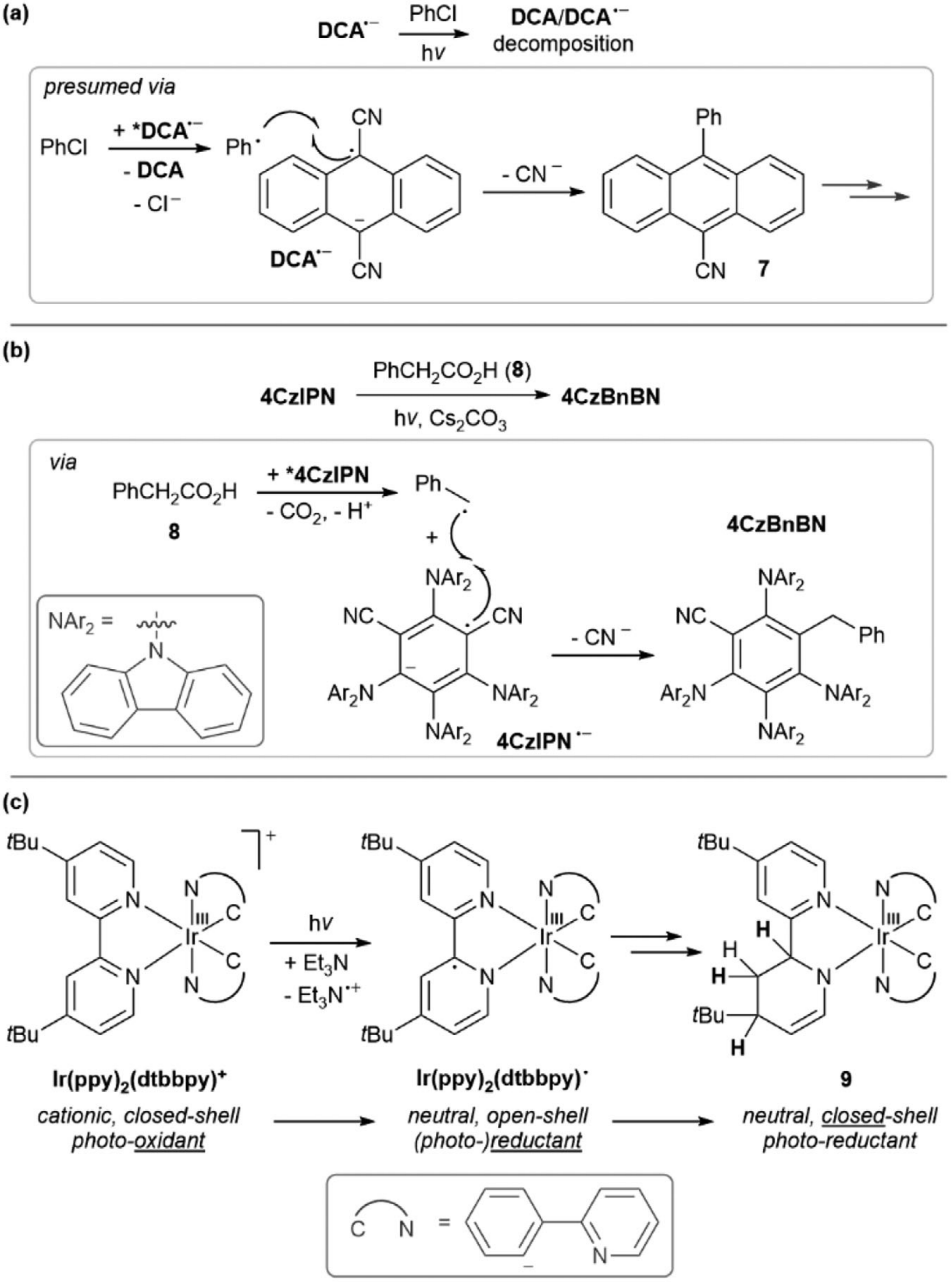
Previously reported in situ transformations of (a) **DCA^•−^**, (b) **4CzIPN^•−^**, and (c) **Ir(ppy)_2_(dtbbpy)^+^**.

As a final example, it is worth noting a series of studies into highly reducing, Ir‐based ‘tandem’ photoredox catalysis performed by the Polyzos group. These described how reduction of challenging substrates by the catalyst **Ir(ppy)_2_(dtbbpy)^+^** can proceed via initial, in situ, photochemical reduction of the dtbbpy ligand to generate a more electron‐rich and therefore more potent photoreductant intermediate, **9** (Scheme 10c) [79, 80, 81, 82, 83]. Although this has generally not been considered a formal example of conPET, the analogy between this mechanism and **PC** ^•−^ decomposition‐based models of conPET reactivity (especially via **PC**•H^−^) is fairly clear.

Interestingly, in contrast to these **PC** ^•−^ cases, to our knowledge analogous degradation pathways have not yet been proposed to account for apparent photoexcited radical reactivity during **PC** ^•+^‐based photo‐*oxidations*, even though essentially identical lifetime arguments should seemingly apply. While this is presumably at least partially attributable to the relative lack of mechanistic studies into oxidative conPET and e‐PRC, it may also reflect the fact that **PC** ^•+^ states tend to be less sensitive than **PC** ^•−^ to trace impurities such as O_2_ or H_2_O, or to other common protic species. Alternatively, the **PC** motifs used so far may simply be inherently more stable. For example, the Lambert group have reported essentially quantitative photocatalyst recovery in reactions using **TAC^+^**, which clearly challenges the idea that such reactions *must* involve catalyst decomposition [22]. On the other hand, some triarylamine‐based **PC** systems (phenothiazines, acyclic triarylamines) can be susceptible to oxidative degradation (e.g. oxidation of S to S═O, carbazole formation) [84, 85].

## 4 Static Quenching via Preassembly/Pre‐Association

### 4.1 Support for conPET/e‐PRC Mechanisms

Despite the strong concerns about photoexcited radical redox reactivity summarized in Sections 2 and 3, synthetic reports during this period continued to frequently invoke the original conPET and e‐PRC mechanisms. In some cases, this was accompanied by a defense of the mechanisms’ feasibility, with this discussion often focusing on the closely related ideas of preassembly/pre‐association and/or static quenching (*vide infra*, Section 4.2).

One of the most prominent challenges to the idea that photoexcited radical redox mechanisms *must* be ineffective began in 2023, with the start of a series of reports from several research groups showing the preparative isolation and reactivity studies of authentic **PC** ^•−^ compounds, including a series of Ir complexes (e.g. **Ir(ppy)_2_(dtbbpy)^•^**) [86]; **DCA^•−^** and **NpMI^•−^** (as [K(crypt)^+^][**PC** ^•−^] salts with robust, weakly interacting counter‐cations; crypt = [2.2.2]cryptand) [74]; **Ru(bpy)_3_^•+^** (as its salt with a weakly interacting BPh_4_^−^ counter‐anion) [87]; **DMQA^•^** [88, 89]; and **Acr^•^** (Figure 3a [33, 58]; note that analogous use of isolated **PC** ^•+^ salts such as [**T*p*BPA^•+^**][PF_6_^−^], [**TCBPA^•+^**][PF_6_^−^], and **PTH^•+^** had also been reported previously [20, 46]; Figure 3b).

**FIGURE 3:**
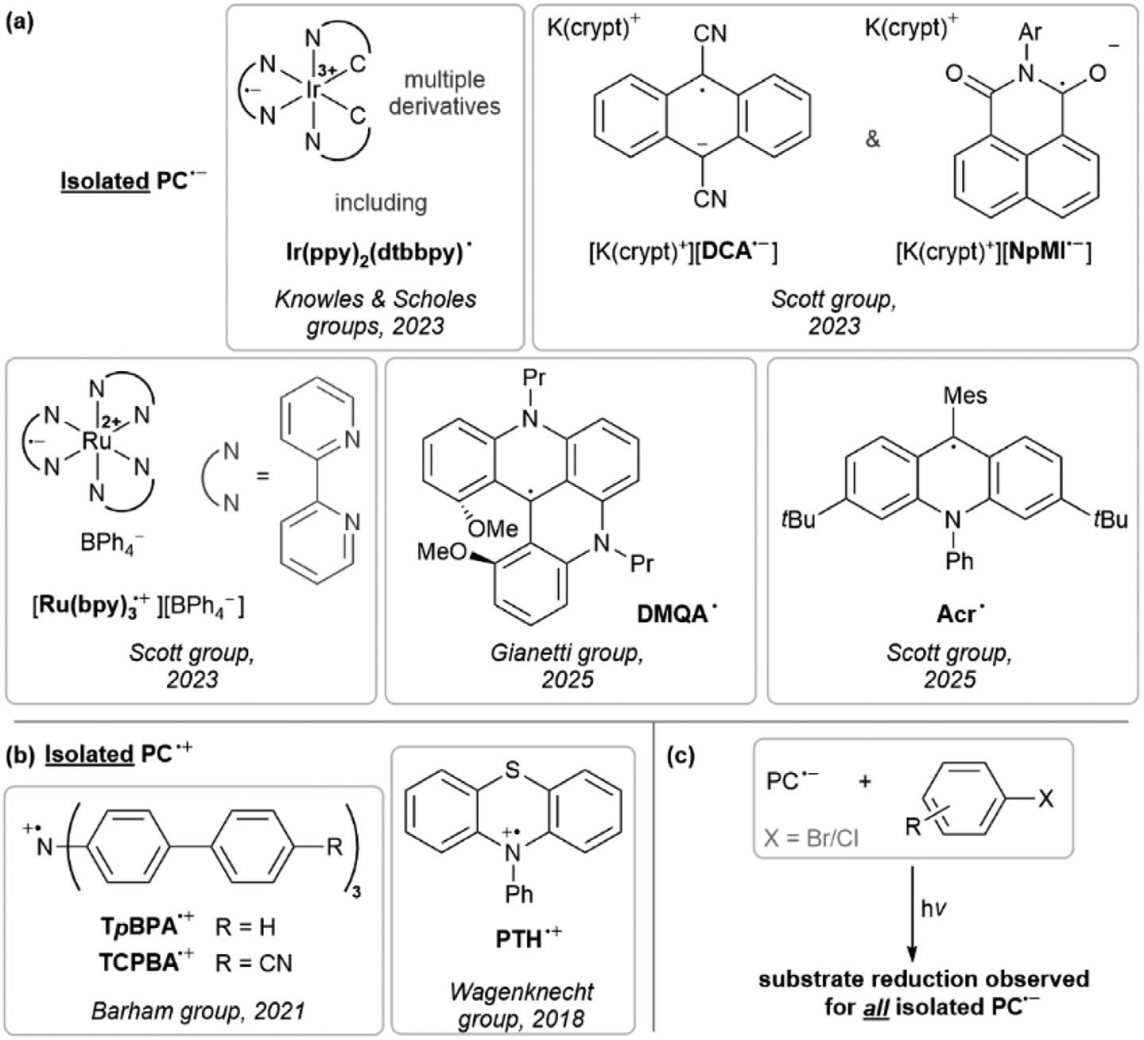
Chemical structures of isolated (a) **PC** ^•−^ and (b) **PC^•+^** states; and (c) observed photoreactivity of isolated **PC** ^•−^ with aryl halides under controlled conditions. Pr = *n*‐propyl.

Isolation of these **PC** ^•−^ states is important, as it allows their photoreactivity to be studied under precisely controlled conditions using relatively simple experimental setups. In comparison with more complex experiments that rely on in situ generation of **PC** ^•−^ from **PC** (as had been the norm previously), these allow for simpler and better‐defined initial sample mixtures, whose results can then be interpreted more easily and with greater confidence (for further discussion, see Section 6.4). Crucially, when these isolated **PC** ^•−^ were irradiated in the presence of synthetically relevant substrates (typically aryl chlorides), reduction of these substrates was *consistently* observed (Figure 3c).

It should be emphasized that each of these results is, on its own, not necessarily mechanistically definitive. For example, in certain cases (but not all) photodecomposition of the **PC**/**PC** ^•−^ motif was observed, meaning that genuine photoexcited radical redox reactivity cannot easily be disentangled from degradation‐based pathways (*cf*. Section 3). Nevertheless, these observations are far easier to reconcile with the original proposals of photoexcited radical redox agent reactivity than with models based on **PC** ^•−^ degradation. This is especially true in cases where there was no evidence of substantial (photo‐)decomposition; where specific, previously‐proposed degradation pathways were unavailable; and where reactivity trends did not align with those of proposed degraded catalysts (*cf*. Figure 2 and discussion in Section 3.1) [90].

Several of these studies also revealed surprising, unexpected trends in reactivity, including complex, substrate‐specific wavelength dependences in many cases. For example, excitation of **NpMI^•−^** (as [K(crypt)^+^][**NpMI^•−^**]) at 530, 630, or 730 nm led to reductive functionalization of methyl 4‐chlorobenzoate (**2**) with very similar efficiency across all three wavelengths (Table 2, top) [74]. This is typical of expectations for a photoinduced reaction. **NpMI^•−^** absorbs similarly at all of these wavelengths and, while each of these excitations may initially generate a different excited state (D_n_), these are all expected to relax to the lowest excited state (D_1_) on ultrafast timescales (usually sub‐ps), which can typically be assumed to be faster than any intermolecular reactivity. As such, photoreactivity should occur via this same D_1_ state in all cases, leading to comparable outcomes (Figure 4) [91].

**TABLE 2:** Relative conversions for reductive functionalization of aryl chlorides by isolated **NpMI^•−^**.^a^[74].

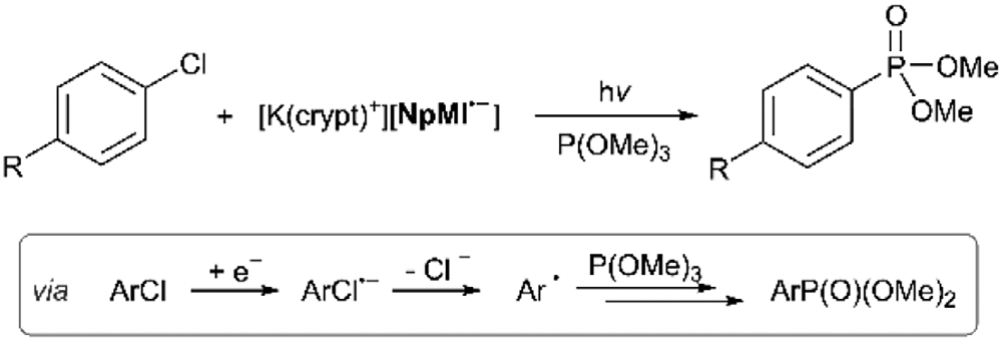				
Normalized conversion to product under:	530 nm	630 nm	730 nm	
R═**CO_2_Me** (**2**)	1.00	1.03	1.12	(**negligible** wavelength dependence)
R═**H**	0.68	0.03	0.00	(**dramatic** wavelength dependence)
R═**OMe** (**6**)	0.64	0.00	0.00	(**dramatic** wavelength dependence)

^a^ Values normalized relative to R═CO_2_Me, 530 nm.

**FIGURE 4:**
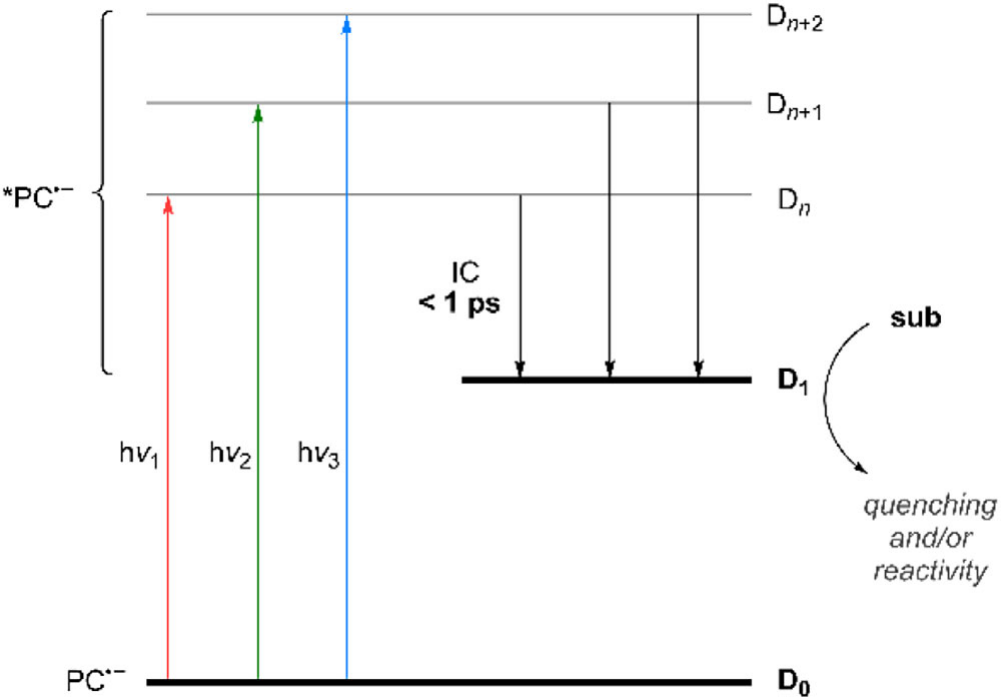
Different excitations of the same **PC** ^•−^ ground state (D_0_) leading to identical photoreactivity via the lowest excited state (D_1_).

In contrast, while reactions with the more challenging to reduce substrates PhCl and 4‐chloroanisole (**6**) led to only slightly diminished performance at 530 nm (*vs*. **2**), reactivity was essentially completely lost for these substrates at 630 or 730 nm, contradicting expectations if reactivity were occurring entirely via D_1_. This obvious wavelength dependence, and its clear accompanying *substrate* dependence, are hard to rationalize via standard photochemical models (*vide infra*; e.g. Section 4.3). It is worth noting that **NpMI^•−^** was observed to be photostable at all three wavelengths (at least under these very controlled conditions), and NMR analysis of model photoreactions suggested efficient regeneration of neutral **NpMI**.

### 4.2 Evidence for the Static Quenching Model

Fundamentally, the bulk photoreactivity observed from isolated **PC** ^•−^ compounds cannot on its own answer the question of *how* substrate reduction takes place, especially in light of what are known to be short *PC ^•−^ lifetimes (*vide supra*). However, as already noted above, one possible answer to this question that had already been suggested is preassembly/preassociation, i.e. an interaction between substrate and **PC** ^•−^ that already exists prior to photoexcitation (Scheme 11). In this scenario, the *PC ^•−^/*PC ^•+^ excited state no longer needs to diffuse to encounter the substrate and as a result SET can occur almost instantaneously, meaning that short excited state lifetimes are much less detrimental. Due to the absence of diffusion, this is known as *static* quenching. Notably, preassembly‐based static quenching has been established for a number of *closed‐shell* **PC** in other photoredox contexts [85, 92, 93, 94], as well as 3d metal complex photoredox catalysts where preassembly has been explicitly proposed to help circumvent short excited state lifetimes [95].

**SCHEME 11:**
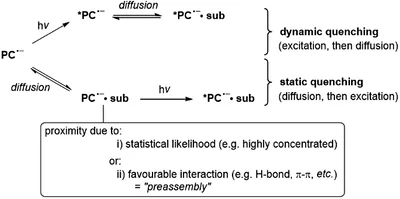
Dynamic vs. static *PC ^•−^/*PC ^•+^ quenching. For simplicity, only the **PC** ^•−^ case is shown.

In the context of photoexcited radical redox, static quenching may result from either of two factors (or both):

- a very high substrate concentration, such that its presence in proximity to **PC** ^•−^/**PC** ^•+^ is simply a statistical likelihood; or
- a specific, energetically accessible ground state interaction between substrate and **PC** ^•−^/**PC** ^•+^ (typically a relatively weak and reversible non‐covalent interaction such as hydrogen bonding or π‐π/π‐T stacking, but in principle also stronger interactions such as Coulombic attraction within an ion pair).

Although the terms ‘preassembly’ and ‘static quenching’ have sometimes been used rather interchangeably in the photoexcited radical redox literature, the former can be more strictly understood to refer specifically to case ii) above, with the latter being a more generic term that covers both options.

#### 4.2.1 Evidence for Preassembly by Steady‐State Spectroscopy and Structure‐Reactivity Trends

In principle, preassembly can be detected using standard steady‐state spectroscopic methods. For **PC** ^•−^/**PC** ^•+^ this could theoretically include new signals, shifts, or other perturbations to UV‐vis, IR(/Raman) and/or ESR spectra, as well as changes to substrate NMR signals. However, in practice, studies to date have rarely reported spectroscopic changes to the **PC** ^•−^/**PC** ^•+^ chromophore upon addition of substrate. This is not necessarily surprising, as these interactions are often proposed to be weak, implying both that any adduct may only be a partial component of an equilibrium mixture, and that adduct formation may not result in substantial electronic (and therefore spectroscopic) changes. For example, as part of our (SJH, DJS) recent investigation of **Acr^•^** photoreactivity towards PhCl, DFT and TD‐DFT calculations suggested that preassembly, though feasible, is likely to be weak (slightly endergonic) and unlikely to result in major UV–vis spectral changes (see Section 4.3) [58].

A rare example where steady‐state spectroscopic changes *were* observed for **PC** ^•+^ in the presence of substrate was reported by us (JPB, DJS) in 2021 [46]. As already briefly alluded to in Section 2.2, excitation of the **PC** ^•+^ states **T*p*BPA^•+^** and **TCBPA^•+^** was observed to facilitate oxidation of even extremely challenging arene substrates (Scheme 12a). Given the extremely short *PC ^•+^ lifetimes (<10 ps) it was proposed that this must occur via static rather than dynamic quenching. Though not explicitly noted in the original report, this would likely be facilitated by the high arene concentrations used in some of these reactions (160 mM–1.95 M). A ca. 10 nm red‐shift in one of the UV‐vis bands of **TCBPA^•+^** was observed in the presence of 700 mM PhCl (from *λ*_max_ = 384 to 395 nm), although no analogous changes were detected upon equivalent exposure of **T*p*BPA^•+^** to mesitylene (despite both **sub**/**PC^•+^** combinations functioning synthetically). More consistent changes were observed by steady‐state ESR spectroscopy, with addition of arene leading to noticeable changes in g value and/or line shape depending on the specific **PC** ^•+^/arene combination. These observations were attributed to preassembly, with this conclusion being supported by DFT and later MD modelling of candidate structures. The former identified π─π and T─π interactions as likely dominant interactions, while the latter predicted an average **PC** ^•+^/**sub** “coordination number” of one, which was proposed to be more consistent with a structured preassembly than with purely statistically governed spatial proximity [46, 61].

**SCHEME 12:**
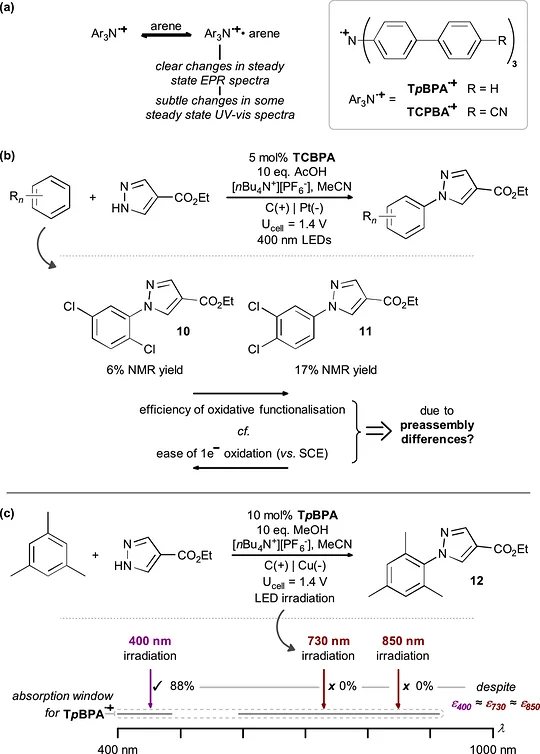
(a) Steady‐state spectroscopic evidence of preassembly for **T*p*BPA^•+^** and **TCBPA^•+^**; and examples of (b) a contra‐thermodynamic efficiency trends and (c) wavelength‐dependent reactivity in corresponding arene oxidation e‐PRC reactions.

Alongside these steady‐state spectroscopic and computational investigations, it was argued that preassembly is also evidenced by other observations, including counterintuitive structure‐activity relationships in reactivity. For example, the synthetic efficiency of oxidation of the isomers of dichlorobenzene followed the trend *ortho* > *para* (by ∼3×; Scheme 12b), despite redox potentials predicting the opposite (i.e. *para* should be *easier* to oxidise). This was attributed to sterically influenced differences in the preassembly's structure and/or thermodynamics, supported by DFT calculations predicting preassembly to be more favourable for the *para* isomer than the *ortho*, in agreement with the observed reactivity trend. Very similar observations were made when comparing *ortho* to *meta* to *para* xylene, and PhMe to PhI, and rationalized via equivalent preassembly‐based models.

Several examples of wavelength‐dependent reactivity were also noted for this system (e.g. Scheme 12c; *cf*. Figure 2) indicating apparent involvement of higher doublet excited states in the observed photoreactivity, i.e. reactivity seemingly requiring excitation to higher excited states, D*_n_*, *n* > 1 (*cf*. similar observations for isolated **PC** ^•−^ discussed in Section 4.1; for more detailed discussion see Section 4.3).

Similar structure‐activity arguments were proposed by us (JPB) and König as evidence for preassembly in *reductive* e‐PRC in the same year [96]. In this case, the reductive C(sp^3^)–O cleavage of phosphinated alcohols (e.g. **13**) was investigated using **NpMI** derivatives. Notably, in several cases where excellent yields were achieved using **^BuO^NpMI** only traces of product (<5% yield) were observed using **NpMI** (Scheme 13a). This is surprising, since the **^BuO^NpMI**/**^BuO^NpMI^•−^** and **NpMI**/**NpMI^•−^** redox potentials are essentially identical (−1.3 V *vs*. SCE), as are the electrogenerated **^BuO^NpMI^•−^** and **NpMI^•−^** UV‐vis spectra, suggesting at first glance that there should be no significant differences in either the generation of the **PC** ^•−^ state or its photoreducing power upon excitation (assuming SET occurs from a ‘bright’ state detectable by UV‐vis). Instead, it was argued that the observed synthetic differences are the result of differences in preassembly (Scheme 13b), possibly due to the differing steric profiles of the *N*‐aryl groups. Again, this conclusion was supplemented by DFT analysis of candidate preassemblies (which predicted significantly stronger binding for **^BuO^NpMI^•−^** than **NpMI^•−^**) and the observation of apparent wavelength dependent, D*_n_* (*n* > 1) reactivity (Scheme 13c).

**SCHEME 13:**
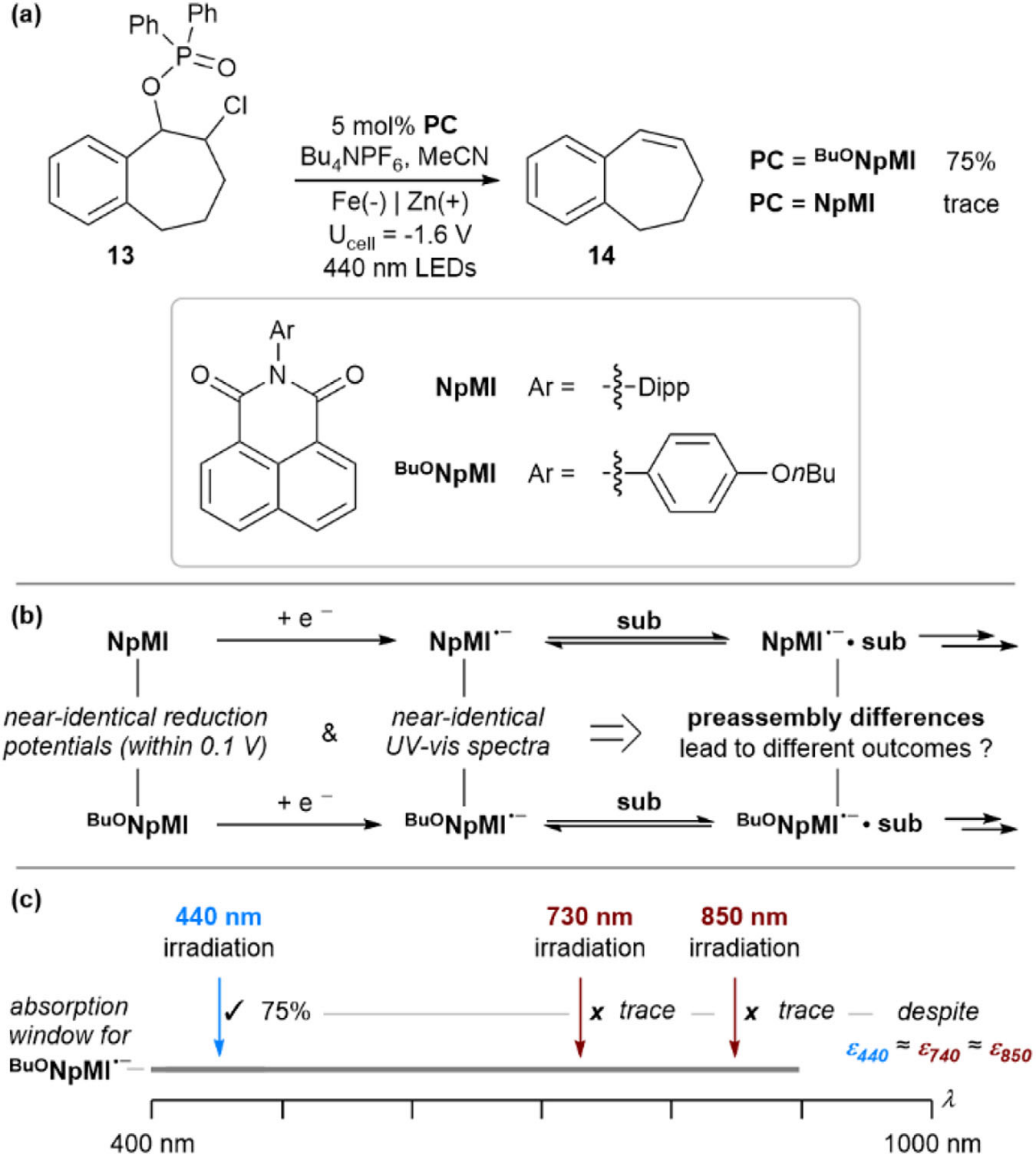
(a) C–O cleavage of a phosphinated alcohol via reductive e‐PRC catalyzed by **NpMI** derivatives, and dramatic differences in performance between **^BuO^NpMI** and **NpMI** despite (b) near‐identical redox and spectral properties; and (c) wavelength‐dependence of the reaction catalyzed by **^BuO^NpMI**.

#### 4.2.2 Evidence for Preassembly by Transient Absorption Spectroscopy

In the absence of characteristic steady‐state features, most investigations of **PC** ^•−^/**PC** ^•+^ preassembly with substrates must instead rely on transient spectroscopic analysis. Broadly speaking, in cases of productive preassembly, the presence of the substrate will be expected to alter the transient photophysical behavior of **PC** ^•−^/**PC** ^•+^ on ultrafast timescales, faster than could reasonably be attributed to diffusion‐limited processes. Such observations necessarily imply an interaction between **PC** ^•−^/**PC** ^•+^ that is occurring faster than the diffusion limit, which requires some form of prior association (i.e. preassembly).

Some of the earliest work in this area was reported by Lee, Cho, You, and colleagues, who used TAS to estimate rates of SET from a pair of donor‐acceptor motif *PC^•−^ states to 4‐bromoacetophenone as being 2–3 orders of magnitude faster than the diffusion limit, although this was caveated by the authors noting that low signal intensities limited the conclusiveness of the observations [97]. The next year, the Hauer group performed TAS investigations into the proposed preassembly of **T*p*BPA^•+^** and mesitylene (*cf*. Scheme 12 and discussion in Section 4.2.1) [60]. Excitation of isolated [**T*p*BPA^•+^**][PF_6_^−^] at 425 nm in DCM in the absence of substrate led to ultrafast changes in UV–vis absorption that could be modelled as a sequential bi‐exponential process with lifetimes of 0.92 ps and 4.8 ps [98]. Though not explicitly stated in the report, this behaviour can presumably be attributed to ultrafast relaxation of an initial, high energy D_n_ state into the D_1_ state (*cf*. Figure 4) [99], followed by slightly slower relaxation of *D*_1_ to the *D*_0_ ground state (Scheme 14a, top). This is supported by good agreement between the 4.8 ps value and the 4.6 ps lifetime measured previously for excitation of **T*p*BPA^•+^** at longer wavelengths (broadband excitation, 490–900 nm) [46].

**SCHEME 14:**
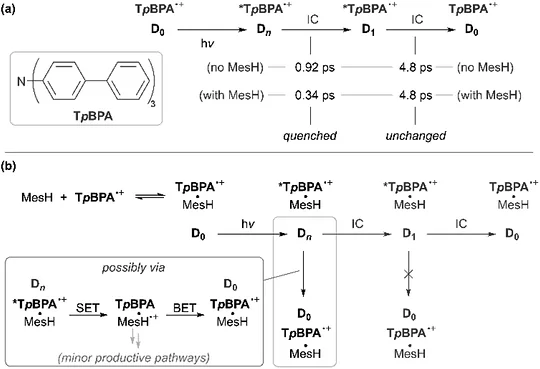
(a) Reported photophysical behaviour of **T*p*BPA^•+^** upon 450 nm excitation in the absence and presence of mesitylene, and (b) possible mechanistic rationale.

Slightly different behavior was observed when this experiment was repeated in the presence of mesitylene, at concentrations (ca. 300 mM, 350 eq. w.r.t. **T*p*BPA^•+^**) similar to those used in synthesis (ca. 175–350 mM arene). While the longer 4.8 ps component was essentially unchanged, the lifetime of the shorter component was found to decrease significantly (from 0.92 to 0.34 ps; Scheme 14a, bottom). This is theoretically consistent with the higher doublet state reactivity proposed for this system: i.e. the substrate is not reactive enough to be oxidized by the D_1_ state but *can* quench the more reactive higher D*_n_* state. Similarly, the fact that a change is being detected within a picosecond is seemingly consistent only with *static* quenching (Scheme 14b). Nevertheless, it should be noted that in this interpretation, productive SET between mesitylene and *T*p*BPA^•+^ must also be very inefficient (although this was not mentioned in the report itself). Even in the presence of mesitylene, the transient UV‐vis changes appear to revert more‐or‐less completely to baseline within a few tens of ps, indicating that *T*p*BPA^•+^ relaxes almost entirely to its ground state, **T*p*BPA^•+^**, rather than forming the neutral SET product, **T*p*BPA**, even in the presence of mesitylene. In other words, while mesitylene may be able to quench the excited state, this quenching mostly appears to provide an alternative mechanism for excited state relaxation to the ground state, such as initial SET being followed by immediate back‐electron transfer (BET) to **sub^•+^**. Productive SET provides at most a much more minor contribution, at least under these conditions (where there is no nucleophilic reagent present to trap **sub^•+^** in competition with BET). This situation can be contrasted with the TAS study of **PDI^•−^** discussed in Section 2.2 (Scheme 5), where for at least some substrates a persistent depletion of the initial ground state **PDI^•−^** signal and concomitant formation of signals attributable to neutral **PDI** were observed (although for more challenging substrates, these persistent features were less evident, suggesting less productive quenching in these cases) [27].

Much more recently, Pfund and Wenger have reported a detailed TAS investigation into the preassembly of radical anion **PC** ^•−^ states, focusing on **DCB^•−^** and **DCT^•−^**, which are somewhat structurally analogous to **DCA^•−^** [100]. Unlike **DCA**, it should be noted that **DCB** and **DCT** have not been used as catalysts in any synthetically focused reports of conPET or e‐PRC. However, as part of their study, the authors did provide proof‐of‐principle demonstration of photocatalytic reactivity proceeding via an apparent photoexcited radical redox pathway. Like **DCA^•−^**, **DCB^•−^** and **DCT^•−^** were found to have extremely short excited state lifetimes (3 ps [33, 51] vs. 4.0 and 1.1 ps, respectively), suggesting that diffusion‐limited photoreactivity is unlikely, and static quenching may occur instead.

To test the latter possibility, two spectroscopic strategies were pursued (note that because isolation of authentic **DCB^•−^** or **DCT^•−^** has not yet been reported, these both required in situ generation of **PC** ^•−^). In the first (which focused especially on **DCT^•−^**), a pump‐pump‐probe method was applied to a mixture of **DCT** and PhNMe_2_ (as a sacrificial reductant) in DMF, with [*n*Bu_4_N^+^][H_2_PO_4_^−^] as an additive to stabilize in situ generated **DCT^•−^** (Figure 5a). In the first pump, a pulse of 355 nm irradiation was used to excite **DCT** and induce a standard photoredox event, to generate the desired **DCT^•−^** (and PhNMe_2_^•+^). After a delay (4 µs) the second, 532 nm pump pulse was then designed to selectively excite **DCT^•−^** only. Meanwhile, the 500 nm probe pulse was used to track the concentration of **DCT^•−^**, which was expected to be the only persistent species with an absorption at this wavelength.

**FIGURE 5:**
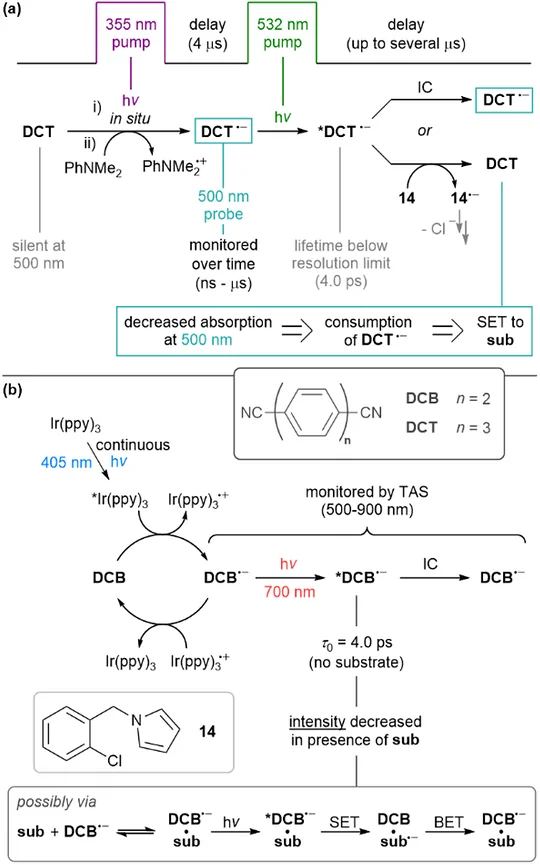
TAS investigation of **DCB^•−^** and **DCT^•−^** preassembly by two different methods. Ir(ppy)_3_ = *fac*‐Ir(2‐(2’‐pyridyl)phenyl)_3_.

This experiment was then repeated in the presence of various concentrations of electron acceptors, such as CH_2_Cl_2_, that do not react with **DCT^•−^** in its ground state. These showed a clear drop in the absorption intensity at 500 nm immediately upon **DCT^•−^** excitation, consistent with photoinduced SET from **DCT^•−^** to the acceptor. By plotting the magnitude of this drop vs. different acceptor concentrations, the authors were able to calculate an SET rate constant of 1.1 × 10^10^ M^−1^ s^−1^ (for CH_2_Cl_2_ as **sub**) which was noted to be 44% higher than the theoretical maximum diffusion‐limited rate constant in DMF (*cf*. Table 1), implying static quenching.

A very similar pump‐pump‐probe strategy has previously been used by Hu, Meyer, and coworkers to investigate *oxidation*: of Cl^−^ by **PTH^•+^** [101]. Again, a bleach of the signal assigned to **PTH^•+^** could be observed following its excitation, directly proportional to the Cl^−^ concentration. Given the short excited state lifetime of *PTH^•+^ (36 ps) the authors argued that this must be a result of static rather than dynamic quenching and were able to estimate an equilibrium constant of 0.36 M^−1^ for the **PTH^•+^**/Cl^−^ preassembly, which in this case can be viewed as a simple ion pairing interaction.

Notably, the analysis in this first method relies on the assumption that SET does indeed occur from the very short lived *PC ^•−^/*PC ^•+^ excited state, rather than any longer lived intermediate (*cf*. Sections 3 and 5). However, the timeframe and resolution of these experiments (ns‐µs) preclude direct observation of any ultrafast (sub‐ns) processes to confirm this. Thus, to support this assumption, Pfund and Wenger's second method (which focused especially on **DCB^•−^**), directly incorporated ultrafast TAS observations [100]. In these experiments a DMF solution of **DCB** and *fac‐*Ir(ppy)_3_ (and [*n*Bu_4_N^+^][H_2_PO_4_^−^] to stabilize in situ generated **DCB^•−^**) was subjected to continuous 405 nm irradiation to generate a steady‐state concentration of **DCB^•−^** (via photoexcitation of Ir(ppy)_3_ and SET from *Ir(ppy)_3_ to **DCB**). While this was ongoing, a 700 nm pump pulse was used to selectively excite this in situ generated **DCB^•−^**, and transient UV‐vis changes between 500 and 900 nm were used to monitor the evolution of the resulting excited state (Figure 5b).

Initial experiments in the absence of substrate showed the expected combination of GSB and excited state absorption (ESA) with baseline recovery (i.e. relaxation of *DCB^•−^ to ground state **DCB^•−^**) occurring with an ultrafast lifetime of 4.0 ps. These experiments were then repeated in the presence of varying concentrations of an aryl chloride substrate (**14**, structure shown in Figure 5b). These showed no change in the excited state lifetime (with baseline recovery of both ESA and GSB), nor were any new spectral features observed (though the initial photoproducts **DCB** and **14** ^•−^ were not expected to absorb within the 500–900 nm spectral window). However, the *intensity* of the transient UV‐vis response was found to decrease as the substrate concentration was increased, which is theoretically consistent with a fraction of the *DCB^•−^ generated undergoing extremely rapid (sub‐ps) quenching, faster than the temporal window of the experiment (ca. 1–100 ps), and too fast for anything other than static quenching (*cf*. Hauer's results with **T*p*BPA^•+^** discussed above, Scheme 14b). Based on this interpretation, the authors were able to use the variation of this intensity change as a function of substrate concentration to estimate an equilibrium constant of ca. 185 M^−1^, and hence a modest free energy of association of ca. 13 kJ mol^−1^.

Though not highlighted in the original report, it is noteworthy that the presence of substrate does not appear to induce a persistent GSB for **DCB^•−^** (i.e. the GSB continues to revert to baseline with the same, unchanged 4.0 ps lifetime as the ESA). This is significant because, much as already noted for Hauer's **T*p*BPA^•+^** study (Scheme 14), it implies a very low net efficiency for SET from *DCB^•−^ to **14**, as quenching appears to primarily regenerate ground state **DCB^•−^** rather than neutral **DCB**. This can be compared with the previous pump‐pump‐probe measurements where, over longer timescales, persistent consumption of **PC** ^•−^/**PC** ^•+^ *is* evidenced.

Some of the most recent evidence in support of preassembly mechanisms has been provided by our own research (SJH, DJS) into the photoreactivity of isolated **Acr^•^** [58]. Like many other isolated **PC** ^•−^, the photoreactivity of **Acr^•^** towards aryl chloride substrates was found to be highly sensitive to factors such as the specific choice of substrate, solvent, and irradiation wavelength. This will be discussed in more detail in Section 5.2. However, while investigating these phenomena several observations were made by ultrafast transient UV‐vis spectroscopy that implied the presence of static quenching. For example, 530 nm excitation of **Acr^•^** in MeCN was observed to result in simple, mono‐exponential behavior, with the *Acr^•^ excited state relaxing to the ground state with a lifetime of approximately 40 ps (Scheme 15a). When this was repeated in the presence of 50 mM 4‐chlorobenzonitrile (**15**) no qualitative difference was observed (including no persistent **Acr^•^** bleach). However, the lifetime was reduced dramatically, to just 22 ps. This indicates additional quenching of *Acr^•^ by the substrate (consistent with reactivity studies) with an efficiency, *η*, of 46%, which is over an order of magnitude greater than the theoretical *η*_max_ for dynamic quenching given by Equation (2) (Section 2.1).

**SCHEME 15:**
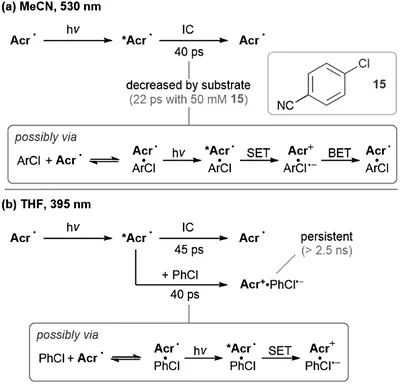
(a) Reported photophysical behavior of **Acr^•^** in the absence or presence of aryl chloride substrates (a) in MeCN under 530 nm irradiation, or (b) in THF under 395 nm irradiation.

It should be noted that despite leading to the same conclusion of preassembly, the observations here are slightly different to those of Hauer (for **T*p*BPA^•+^**, Scheme 14) or Pfund and Wenger (for **DCB^•−^**/**DCT^•−^**, Figure 5). Unlike the former, a difference in lifetime for the *D*_1_ *PC ^•−^ state (not D_n_) was observed, which is also unlike the latter where merely a difference in *intensity* of the D_1_ transient was observed (Figure 6). This would seem to indicate somewhat slower SET kinetics following initial **Acr^•^** excitation, sufficient for this step to become experimentally resolvable (ps rather than sub‐ps timeframe). Slightly different behavior again was observed when **Acr^•^** was instead excited at 395 nm in THF (Scheme 15b). In the absence of substrate, a mono‐exponential response very similar to that for 530 nm excitation in MeCN was observed, with a 45.0 ± 0.5 ps *Acr^•^ lifetime. However, in the presence of 50 mM PhCl a bi‐exponential response was observed, with the lifetime of the initial excited state decreasing to 40.4 ± 0.6 ps and, rather than relaxing smoothly to the ground state, instead evolving into a second, much longer‐lived state (persisting well beyond the 2.5 ns experimental window) that was presumed to correspond to the initial post‐SET product, i.e. **Acr^+^** •PhCl^•−^. Again, the magnitude of the change in the initial *Acr^•^ lifetime suggests quenching by PhCl with a lifetime that is substantially higher (by ca. 2x) than the maximum predicted by Equation (2), thus again implying static quenching (with the feasibility of preassembly via T─π interactions also being supported by DFT calculations). The reason why the initial photoproduct should be so much more persistent in this case than in others currently remains unclear (and no significant bulk change was observed over the course of the experiment).

**FIGURE 6:**
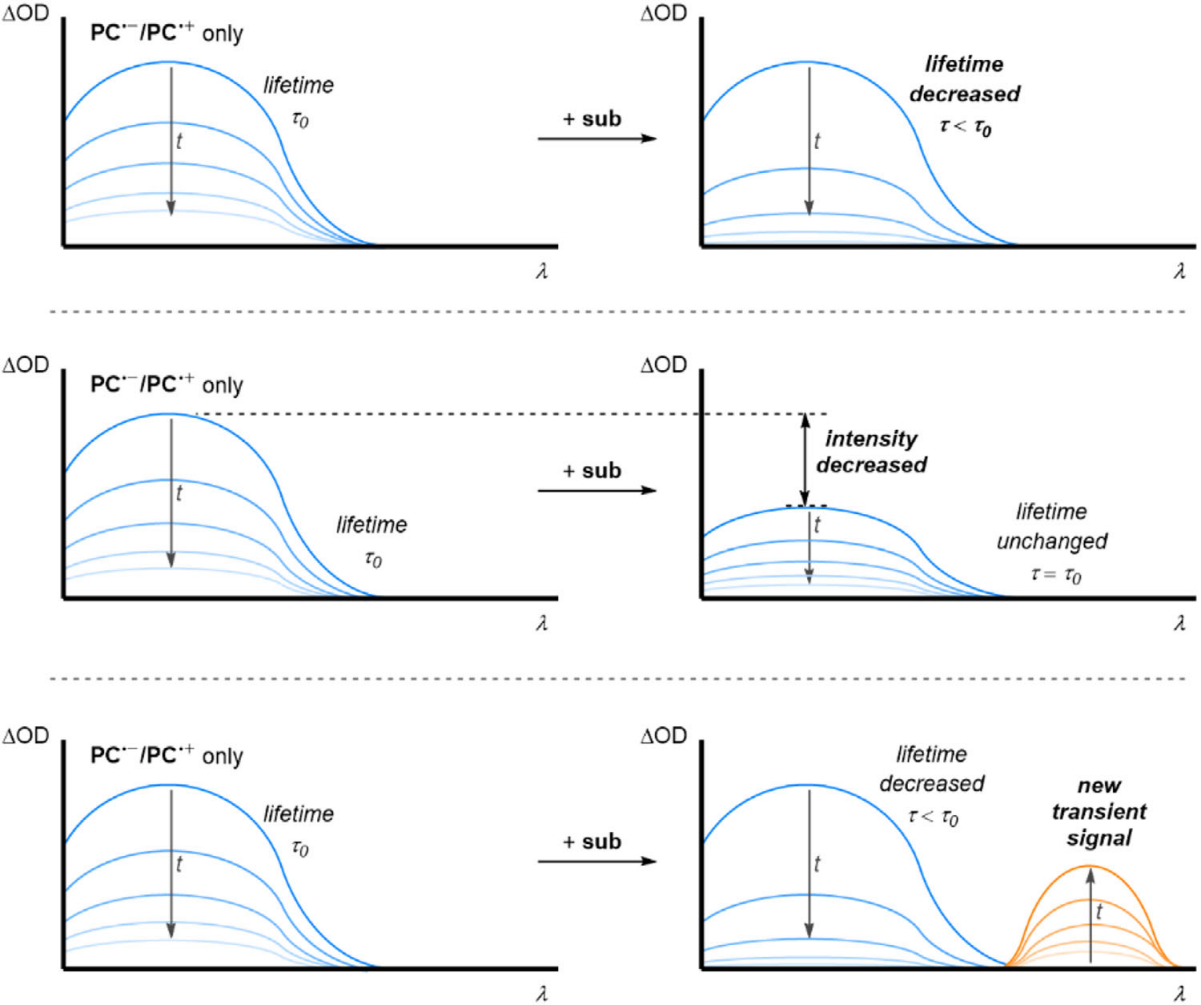
Different TAS behaviors taken as evidence of static quenching in different studies of photoexcited radical redox reactivity.

#### 4.2.3 Inference in Static Quenching Observations

At this point, it is worth explicitly emphasizing that, in contrast to the key intermediates proposed in other mechanistic rationales, evidence for **PC** ^•−^/**PC** ^•+^ preassemblies with substrates generally relies on *inference* rather than direct observation. The **PC** ^•−^ derivatives discussed in Section 2, such as **PC**•H^−^, are easily detectable by standard steady‐state methods such as NMR or UV‐vis, and the solvated electrons that are the focus of Section 5 show characteristic near IR (NIR) traces during TAS experiments. However, relevant preassemblies most often lack clear and convenient spectroscopic signatures that can be easily resolved from those of their constituent **PC** ^•−^/**PC** ^•+^ and substrate (*cf*. Section 4.2.1). In the absence of *direct* observation, arguments for preassembly must therefore typically rely on indirect evidence related to phenomena such as quantum yields and diffusion lifetimes. While much effort has been made by numerous researchers (including several of us) to ensure that they are robust, it must be acknowledged that these indirect arguments are inevitably more vulnerable to mistaken assumptions or unexpected, unaccounted‐for phenomena. This can be exacerbated by the need for relatively complex experimental designs using non‐routine equipment (e.g. TAS).

Nevertheless, despite this note of caution, it can also be re‐emphasized those multiple studies have now reported *substrate‐dependent* changes in **PC** ^•−^/**PC** ^•+^ photophysical behavior occurring on ultrafast timescales, seemingly faster than the diffusion limit (Section 4.2.2), while several studies have also observed substrate/**PC** selectivity/reactivity relationships that are misaligned with conventional expectations (e.g. based on redox potentials). *For now*, only preassembly models have been able to confidently account for these observations.

### 4.3 Higher Doublet State Reactivity in the Static Quenching Model

One particularly interesting strength of the preassembly model is its potential ability to account for otherwise highly unusual photoreactivity apparently occurring via higher doublet excited states (D*_n_*, *n* > 1). As has already been noted at several points in sections 4.1 and 4.2, there have been many examples in the field of photoexcited radical redox where observed reactivity is found to be highly—and counterintuitively—wavelength dependent, both in bulk synthetic reactions and in more controlled mechanistic studies. In particular, superior reactivity is frequently observed when **PC** ^•−^/**PC** ^•+^ are excited with higher energy photons, even if they absorb equally well at longer wavelengths (and these differences are generally too dramatic to be attributable to differences in LED output power; see Section 6.5).

As already noted, this is generally inconsistent with conventional dynamic quenching models (*cf*. Figure 4). While excitation at different wavelengths may *initially* generate different D_*n*_ excited states, these should typically relax to the D_1_ state incredibly quickly (usually < 1 ps). This relaxation should outcompete any diffusion‐limited steps and thus force all observable photoreactivity to occur through the same D_1_ state, regardless of the initial excitation wavelength (at least in the absence of any photoinduced rearrangements or other processes occurring *intra*molecularly).

**PC** ^•−^/**PC** ^•+^ decomposition‐based models also struggle to consistently account for observed wavelength dependence. In some cases, wavelength dependence may be attributable to degradation products only absorbing across a more limited set of wavelengths than the original **PC** ^•−^/**PC** ^•+^ (Scheme 16, top), or to relevant photodecomposition only being induced by high energy photons (Scheme 16, bottom; e.g. **NpMI^•−^** is photostable in MeCN under 530, 630, or 730 nm irradiation, but not 455 nm) [74]. However, as has already been discussed in Section 4.1, in practice this wavelength dependence often does not correlate well with the absorption spectra of likely degraded **PC** states (*cf*. Figure 2), and/or occurs even under conditions where degradation is unlikely.

**SCHEME 16:**
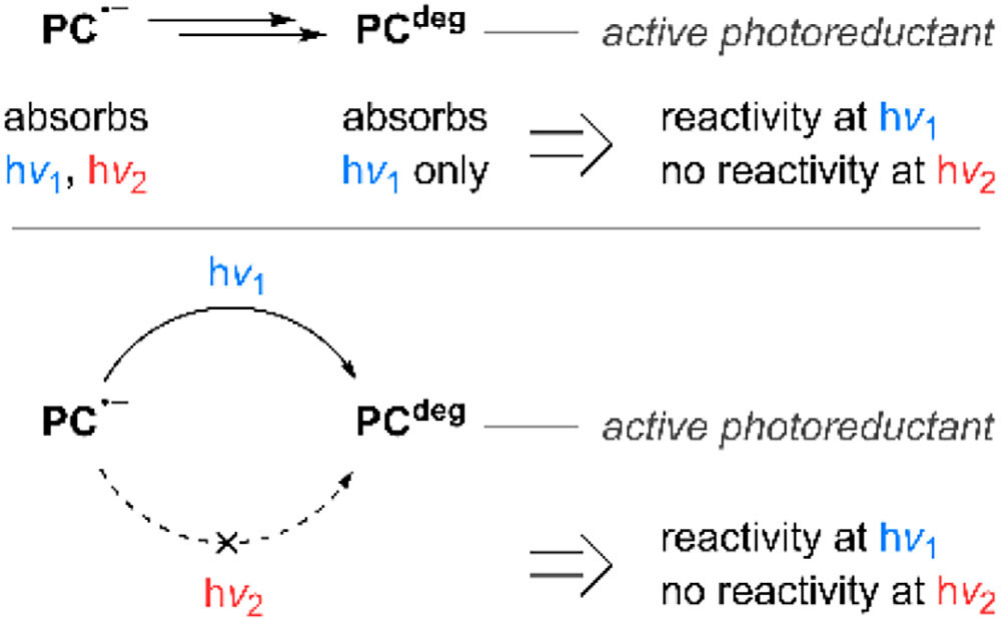
Possible explanations for wavelength dependence in apparent **PC** ^•−^ photoexcited radical reactivity actually mediated by degradation products, **PC** ^deg^.

Current solvated electron models suffer similar limitations when trying to fully explain these wavelength‐dependent observations, as will be discussed in more detail in Section 5.3.

In contrast, preassembly offers a potentially very clear explanation. Just as static quenching models allow the D_1_ state to overcome a short lifetime by bypassing diffusion (Scheme 11), they might also do the same for even shorter‐lived, higher D*_n_* states (Scheme 17). This rationale was initially invoked by Miyake (in 2020) [64] and then us (JPB, DJS in 2021) [46, 96] to account for surprising wavelength dependence observations in a number of early synthetic and mechanistic studies (see Sections 4.1 and 4.2.1). However, these proposals were speculative, without support from direct spectroscopic observations (though *cf*. Hauer's observations summarized in Scheme 14). Indeed, it was uncertain whether even preassembly would induce sufficiently rapid excited state SET to compete with the exceedingly rapid relaxation of most higher D*_n_* states to D_1_.

**SCHEME 17:**
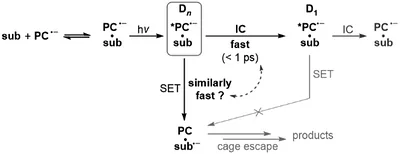
Higher doublet state reactivity mediated by preassembly. For simplicity, only the **PC** ^•−^ case is shown.

Possibly the first explicit spectroscopic observation of a **PC** ^•−^ state responding differently to the presence of substrate on ultrafast timescales *depending on excitation wavelength* was provided during our (SJH, DJS) investigations of isolated **Acr^•^** [58]. As already noted in Section 4.2.2, the presence of PhCl during 395 nm irradiation of **Acr^•^** in THF leads to rapid (tens of ps) emergence of a new, long‐lived transient state assigned as the initial photoproduct (Scheme 15b). In contrast, no substantial change in the presence of PhCl was observed during equivalent experiments using 530 nm irradiation. This correlated well with reactivity studies showing that **Acr^•^** can effect productive reduction of PhCl under 395 nm irradiation but not 530 nm (Scheme 18a), and was further supported by TD‐DFT calculations. Specifically, it was shown that for a model preassembly between **Acr^•^** and PhCl, the presence of the PhCl leads to the formation of two new excited states very close in energy to one another that can be characterized as ‘charge transfer to PhCl’ states, i.e. **Acr^+^** •PhCl^•−^, consistent with the expected initial photoproduct (Scheme 18b). These new transitions have very low calculated oscillator strengths, so are unlikely to be populated by excitation directly. However, their energies, which correspond to photons of ca. 460–470 nm, are well‐placed for them to be accessible via relaxation of a higher 396 nm excitation, while being energetically inaccessible upon lower energy 530 nm excitation.

**SCHEME 18:**
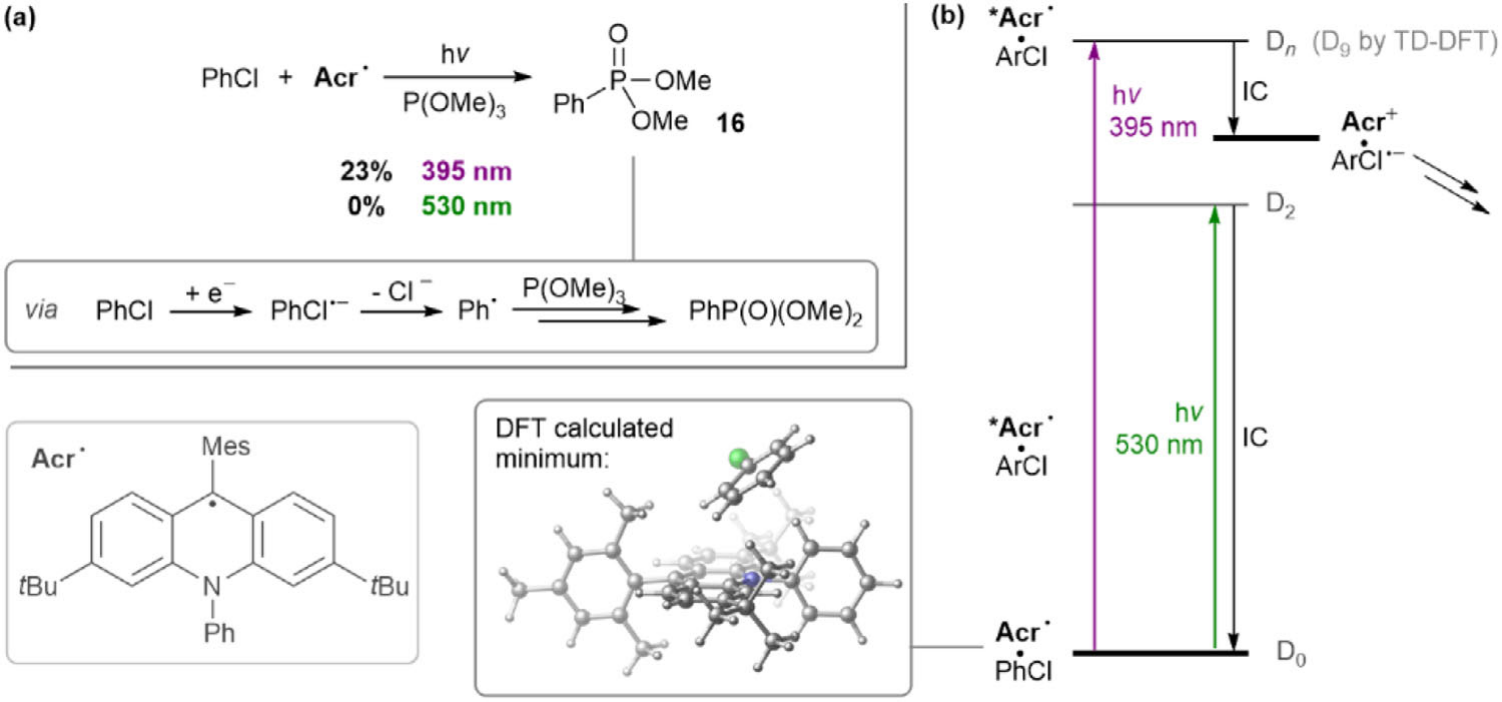
(a) Wavelength‐dependent photoinduced reduction of PhCl by **Acr^•^** in THF, and (b) mechanistic rationalization based on the calculated electronic structure of an **Acr^•^** •PhCl preassembly [58].

More recently, a much more comprehensive spectroscopic investigation of D*_n_* (*n* > 1) reactivity has been reported by Pfund and Wenger, focusing on **DCT^•−^** [31, 102]. This new study employed a very similar pump‐pump‐probe method to the one previously used by the same authors to investigate the involvement of **DCT^•−^** in preassembly‐based static quenching work (*cf*. Figure 5a and Section 4.2.2) [100]. An initial 355 nm pump pulse was again used to generate **DCT^•−^** in situ in DMF, via photoinduced SET from PhNMe_2_ to *DCT (Figure 7). After 2 µs, the second pump pulse was then designed to selectively populate either the *DCT^•−^ D_1_ or D_2_ state, using either 1064 nm or 532 nm photons, respectively. Meanwhile, the probe pulse was used to track the concentration of **DCT^•−^** throughout the several µs experimental window, via its absorption at 500 nm (as in the previous study, it was assumed that no other persistent species were likely to absorb at this wavelength).

**FIGURE 7:**
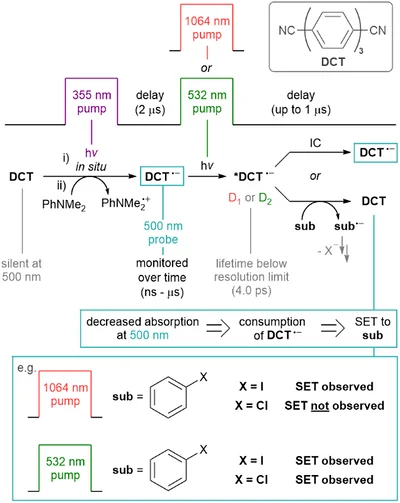
TAS investigation of **DCT^•−^** D*_n_* (*n* = 1, 2) photoreactivity.

This was then repeated in the presence of various aryl halide substrates, in varying concentrations. As with the previous study, a drop in **DCT^•−^** concentration immediately following the second pump pulse was taken as evidence of a photoinduced SET event (presumed to be via preassembly, based on the conclusions of the authors’ previous work). As expected, under 1064 nm excitation this drop was only observed for relatively easily reduced substrates (at least under a mono‐photonic regime), with more challenging aryl chlorides producing no response, consistent with their reduction potentials being too negative for the *DCT^•−^ (D_1_)/**DCT** couple. However, in contrast, even much more challenging substrates such as PhCl could react under the higher energy 532 nm excitation, fully consistent with the idea of reduction of these substrates occurring from the D_2_ state of *DCT^•−^ but not the D_1_.

As expected, there was a strong correlation between the efficiency of **DCT^•−^** oxidation and the electronic structure of the substrate, with easier‐to‐reduce substrates leading to greater reactivity. Nevertheless, the authors noted that the exact shape of this relationship is somewhat complex, which was elegantly rationalized by using Marcus theory to account for relative SET rates (which reach a maximum when the thermodynamic driving force for SET matches the reaction's reorganization energy but are slower when this driving force is significantly smaller or higher; for a more detailed description, readers are encouraged to refer to the original publication [31]). Even so, it should be noted that the magnitude of the measured response is likely to be affected by the electronic structure of the substrate in other ways that are not explicitly considered by Marcus analysis alone. For example, the degree of **DCT^•−^** oxidation that persists on the ns‐µs experimental timescale will depend not only on the rate of initial SET from *DCT^•−^ to the aryl halide (ArX), but also on the relative rates of subsequent productive ArX^•−^ loss (via fragmentation to Ar^•^ and X^−^ and/or escape from the **DCT** solvation shell) and unproductive BET from ArX^•−^ to **DCT** (which regenerates **DCT^•−^**). Both of these pathways are likely to be highly sensitive to the ArX electronic structure (*cf*. Scheme 24b, below). Similarly, it is likely that different ArX may display substantially different thermodynamic affinities for initial preassembly with **DCT^•−^** (*cf*. Section 4.2.1) [96].

## 5 Reductive Photoexcited Radical Redox via Solvated Electrons

### 5.1 Initial Observations of Solvated Electrons

Alongside preassembly, the other currently most prominent rationale for *reductive* photoexcited radical redox reactivity (that does not require degradation of the **PC** structural motif) is the intermediacy of solvated electrons, e^−^_(solv.)_ (Scheme 19a). Solvated electrons are familiar to almost all chemists as extremely powerful reductants. However, for most practitioners, consideration of e^−^_(solv.)_ is associated with specific, highly polar (mostly protic) solvents such as NH_3_ (e.g. traditional Birch reduction) and H_2_O. As such, early consideration of the possible role of e^−^_(solv.)_ in conPET‐type reactivity was largely focused on (semi‐)aqueous systems. Thus, early synthetic reports of conPET‐type catalysis by Ir‐ and Ru‐based **PC** in either purely aqueous or aqueous micellar solutions were reported by the groups of Goez [103, 104, 105, 106], Kerzig and Wenger [107, 108], and König [109], with these reports each proposing photoinduced release of a hydrated electron, e^−^_(aq.)_, as a likely key step. These proposals were generally supported by indirect spectroscopic or other experimental observations (e.g. use of chemical electron traps), with Kerzig and Wenger also reporting direct detection of the characteristic NIR spectroscopic signature of e^−^_(solv.)_ in TAS experiments (*vide infra* in this Section).

**SCHEME 19:**
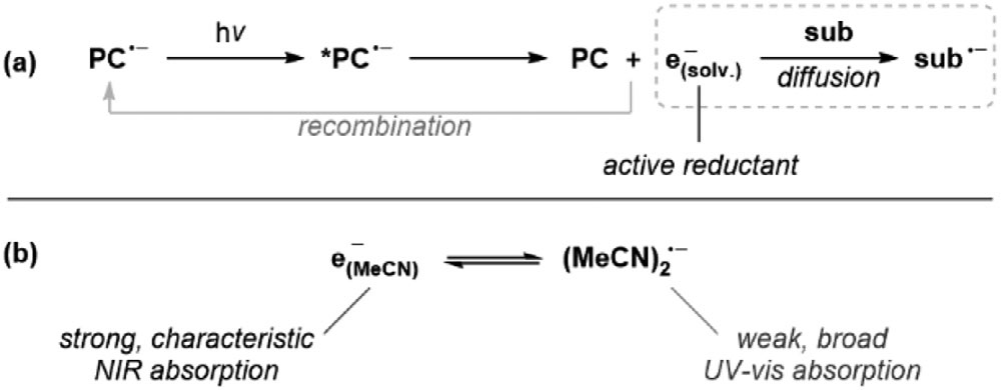
(a) Photoexcited radical redox reduction of a generic substrate via solvated electron formation, and (b) equilibration between a cavity solvated electron and reduced solvent dimer in MeCN.

In contrast, it is only much more recently that consideration of photoexcited radical redox potentially occurring via e^−^_(solv.)_ has commonly been extended to the more conventional organic solvents that are more typical for conPET, e‐PRC, and related reactions (although initial evidence using flavin derivatives, including transient NIR observations, was presented by Cibulka and colleagues as early as 2022 [110]). Pioneering work in this area was provided in 2024 by a team led by Trosi, Ventura, and Ceroni, who studied the **PC** ^•−^ states derived from two closely related TADF‐type photocatalysts, **4CzIPN^•−^** and **4DPAIPN^•−^**, in MeCN [45]. Though less common than hydrated or ammonia‐solvated electrons, electrons solvated by MeCN are known. They exist in an equilibrium between a cavity solvated form, e^−^_(MeCN)_, and a dimeric reduced form of the solvent, (MeCN)_2_^•−^, with this equilibrium being established on a *ca*. 80 ps timescale [111], and slightly favouing e^−^_(MeCN)_ in pure MeCN (*K* ≈ 0.44) [112]. The reduced solvent dimer (MeCN)_2_^•−^ is known to give a weak, broad UV‐vis absorption in the visible region, while e^−^_(MeCN)_ gives a much stronger and more characteristic absorption in the NIR with a maximum around 1400–1500 nm (Scheme 19b). Because the latter feature is typically easier to detect, most of the discussion herein will focus on e^−^_(MeCN)_. However, in practice, both halves of the equilibrium can likely act as competent reductants.

TAS analysis of **4CzIPN^•−^** (generated in situ via reaction with [*n*Bu_4_N^+^]_2_[C_2_O_4_^2−^] under 390 nm irradiation, Scheme 20) upon excitation at 480 nm did indeed reveal a broad NIR band with a maximum at 1440 nm [45]. This was accompanied by transient spectral changes in the UV–vis region. However, these two sections of the spectral window were observed to change over very different timescales, consistent with the expectation that they represent different species. Specifically, the transient signal in the visible region was observed to decay to zero via an ultrafast bi‐exponential process with lifetimes of ca. 300 fs and 27 ps. These presumably correspond to relaxation of the initially generated D*_n_* excited state first to D_1_, and then from D_1_ to the D_0_ ground state. In contrast, the NIR signal assigned to e^−^_(MeCN)_ was observed to display more complicated behavior, modelled as a multi‐exponential decay with component lifetimes varying from hundreds of ps to several ns. This hints at relatively complex dynamic behavior for the recombination e^−^_(MeCN)_ and **4CzIPN** but also indicates that recombination can be slow enough for diffusion‐limited e^−^_(MeCN)_ capture by a substrate to theoretically become competitive.

**SCHEME 20:**
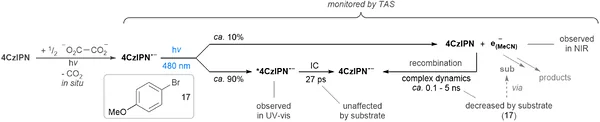
TAS observation of e^−^_(MeCN)_ formation upon photoexcitation of in situ generated **4CzIPN^•−^** in MeCN.

Based on the intensity of the e^−^_(MeCN)_ NIR signal (and previous reports of its extinction coefficient), it was estimated that its formation occurs with ca. 10% quantum yield which, while not particularly high, is synthetically reasonable. It can be noted that, in principle, this 10% e^−^_(MeCN)_ formation should be accompanied by a correspondingly persistent 10% formation of neutral **4CzIPN**, alongside an equivalent bleach of the original **4CzIPN^•−^**. Unfortunately, any such features were not explicitly noted in the report, and their presence or absence is not clear from the data as presented. However, it is possible that reliable deconvolution of such a minor component from the overall TAS data may not be possible.

Consistent with the proposed role of e^−^_(MeCN)_ as an active reductant, repeating the above TAS measurements in the presence of increasing concentrations of an aryl halide substrate (4‐bromoanisole, **17**) led to a steady decrease in the persistence of the observed e^−^_(MeCN)_, but no change in the *4CzIPN^•−^ D_1_ lifetime. This is in line with expectations that the substrate should interact with the former but not the latter.

As well as **4CzIPN^•−^**, this initial study also showed essentially equivalent behavior for **4DPAIPN^•−^**, this time upon photoexcitation at 600 nm. In a subsequent correspondence the same team extended this analysis to another close structural analogue [113]. As an aside, it is worth noting that in both of these reports the authors identified previous mistaken attributions of emission to the (actually non‐emissive) **PC** ^•−^ states involved, again highlighting the risks of in situ **PC** ^•−^ characterization (although this apparent misattribution has itself been challenged by the authors of one of the original synthetic reports) [114].

It can also be noted that during these TAS investigations of **4CzIPN^•−^** and **4DPAIPN^•−^** very high excitation pulse energies were used, as high as 8 µJ/pulse [45]. Energies below 1 µJ/pulse are more typical in such experiments (assuming typical focusing conditions of 100–200 µm) to minimize the likelihood of two‐photon ‘double excitation’ processes. Such processes are generally precluded when considering synthetic studies, due to the limited intensity of the light sources employed (e.g. LEDs) [108, 115]. However, the much greater intensity of laser light sources, and especially fs‐*pulsed* lasers used in TAS (which by design provide bursts of photon energy within very narrow time windows, ∼100 fs), makes simultaneous absorption of two photons by one molecule much more feasible. One‐ and two‐photon excitations can usually be distinguished by repeating experiments at lower pulse energies; however, it is not specifically stated whether such experiments were performed in this case. High pulse energies may also lead to undesired photobleaching in some systems [116].

### 5.2 Extension to Other PC^•−^ Motifs

In parallel with Ceroni and coworkers’ studies of TADF chromophores, MeCN solvent reduction was also considered by the Gianetti group for an isolated helicene radical **PC** ^•−^ state, **DMQA^•^** [89]. However, the authors’ eventual 2025 report ultimately proposed formation of e^−^_(MeCN)_ to be inviable, based on a failure to observe its characteristic transient NIR signature. Instead, substrate reduction was tentatively proposed to occur via formation of (MeCN)_2_^•−^ as the active reductant, although given the rapid equilibration expected between this and e^−^_(MeCN)_, the question of how one might form in the absence of the other is unanswered.

Nevertheless, more confident extrapolation of the solvated electron model to other **PC** motifs has recently been described by several research groups. In the broadest of these studies, the Nocera group have reported that the transient NIR signal associated with e^−^_(MeCN)_ can also be observed upon sufficiently short wavelength excitation of isolated **DCA^•−^**, **NpMI^•−^**, and **Acr^•^** (Scheme 21a) [33]. For **DCA^•−^** it was noted that this is accompanied by formation of neutral **DCA**. It was also shown that the lifetime of the e^−^_(MeCN)_ signal diminishes as PhCl is added, with quenching apparently close to the diffusion limit. This is consistent with reduction of PhCl upon photoexcitation of **DCA^•−^** previously observed by us (SJH, JPB, DJ [74]. Similarly, the Nicewicz group had also shown shortly beforehand that 400 nm excitation of **Acr^•^** (in that case generated in situ from **Acr^+^** and Et_3_N under 390 nm irradiation) leads to a transient NIR signal consistent with e^−^_(MeCN)_, whose decay is accelerated by addition of a substrate (acrylamide; Scheme 21b) [117].

**SCHEME 21:**
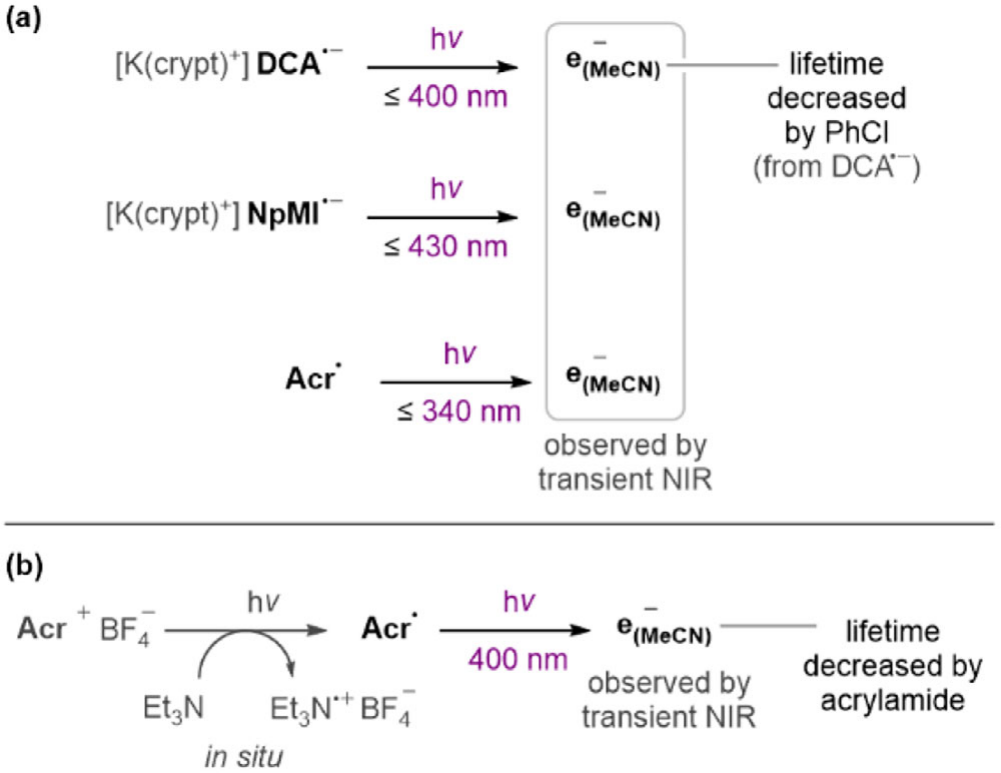
TAS observation of e^−^_(MeCN)_ formation upon photoexcitation in MeCN of (a) various isolated **PC** ^•−^ and (b) in situ generated **Acr^•^**.

Although most studies of e^−^_(solv.)_ pathways have relied on transient NIR methods to observe the key intermediates directly, their existence can also sometimes be inferred from other observations. As already discussed in Sections 4.2.2 and 4.3, our (SJH, DJS) investigations of isolated **Acr** ^•^ suggested that in THF solutions and/or under 530 nm excitation, photoexcited radical redox reduction of ArCl substrates can occur via a preassembly‐based static quenching mechanism (*cf*. Schemes 15 and 18). Nevertheless, it was noticed that greatly improved rection performance was achieved when performing reactions in MeCN and at a shorter wavelength (395 nm) (Scheme 22) [58]. This combination was also found to lead to substantially more complex photophysical behaviour for **Acr^•^**, with a reproducible, long‐lived (*ca*. 10 ns) emission being detected, alongside an additional, similarly persistent trace in the transient UV‐vis spectrum. After close inspection of the emission, excitation, and TAS spectra, it was possible to assign all of these new features to *Acr^+^, i.e. to in situ formation of a steady‐state concentration of oxidized **Acr^+^** (*cf*. Scheme 6). This formation was noted to be reversible (no persistent, bulk photodecomposition of **Acr^•^** to **Acr^+^** or other species) and to occur even in the absence of any other solute (e.g. a substrate), implying that it *must* be accompanied by transfer of the missing electron to the solvent, i.e. formation of e^−^_(MeCN)_/(MeCN)_2_^•−^. This would then provide a second pathway for aryl chloride reduction, alongside preassembly (Scheme 22b). Notably, this pathway is not expected to be available either in THF (which is less polar and less able than MeCN to stabilize a ‘free’ electron and/or be reduced) or under 530 nm excitation (where photons lack sufficient energy to eject an electron; see Section 5.3 for further discussion).

**SCHEME 22:**
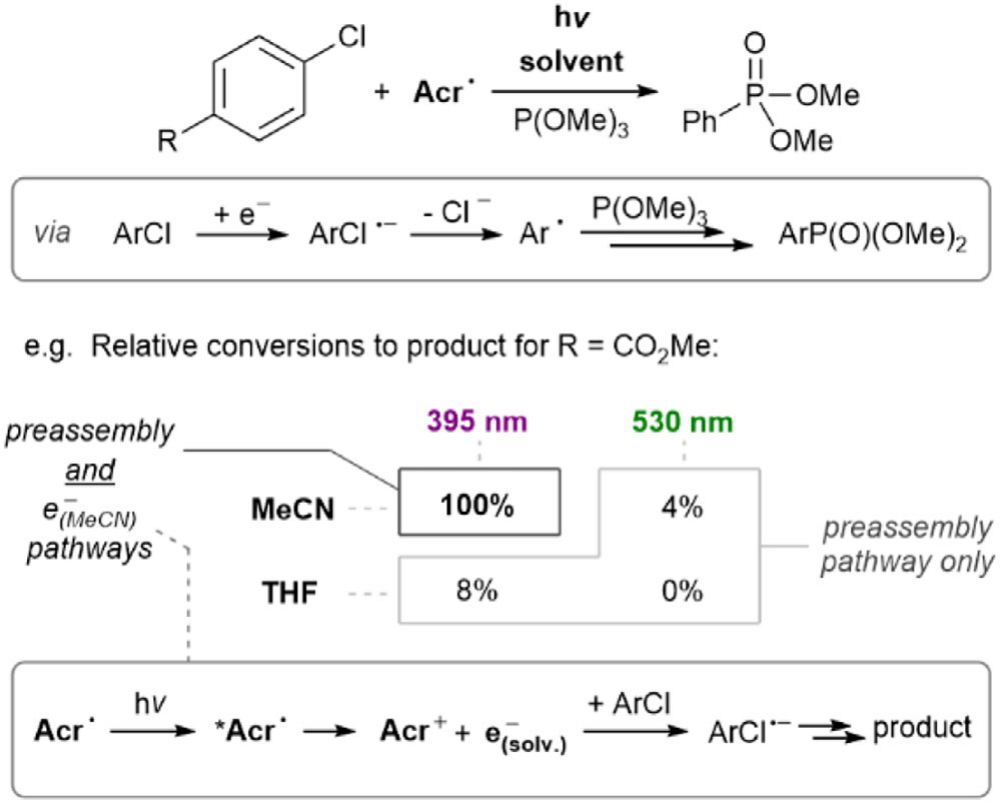
Improved photoinduced reduction of aryl chlorides by **Acr^•^** in MeCN under 395 nm irradiation and rationalization through existence on a second mechanistic pathway. Conversions have been normalized relative to 395 nm in MeCN.

A similar inference has also been used to suggest reduction of several solvents (MeCN, DMSO, CH_2_Cl_2_) upon photoexcitation of in situ generated **Rh6G^•^**, based on observation of TAS UV‐vis signatures consistent with its oxidation to **Rh6G^+^** [118, 119].

### 5.3 Solvent and Wavelength Dependence in Solvated Electron Models

As alluded to in Section 5.2, solvated electron‐based models provide a convenient explanation for the significant sensitivity displayed by many (apparent) photoexcited radical redox reactions to the choice of solvent. In particular, several reports have noted reactions that work well in MeCN either becoming much less efficient [58] or shutting down entirely [117] in other solvents, which can be attributed to these solvents’ less ‘reducible’ nature (or lower stability upon reduction) and correspondingly weaker ability to facilitate formation of e^−^_(solv.)_.

Similarly, these models also provide a simple explanation for observed wavelength dependences (*cf*. Section 4.3), at least in the broad strokes (Scheme 23a). Ejection of an electron into the solvent is generally expected to be possible only for photons above a certain threshold energy (which will depend on **PC** ^•−^ structure, being lower for more strongly reducing ground states), with lower energy photons leading only to more conventional, localized excited states. For example, based on their transient NIR observations, (Scheme 21a) the Nocera group have reported that electron detachment from **NpMI^•−^** requires photon energies corresponding to a maximum photon wavelength of approximately 430 nm [33].

**SCHEME 23:**
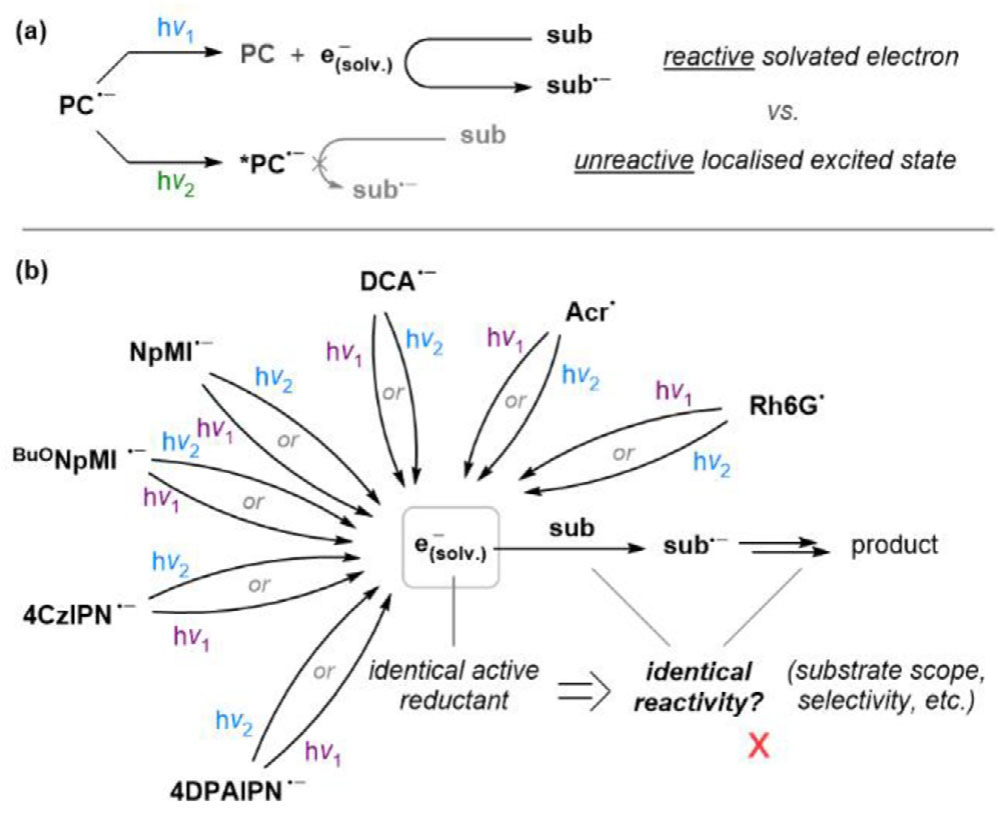
(a) A partial explanation for wavelength‐dependent photoexcited radical redox reductions via solvated electron formation, and (b) a current limitation of this explanation.

Nevertheless, it should be noted that unlike preassembly models, current understanding of e^−^_(solv.)_ models cannot yet account for *all* wavelength‐dependent phenomena. For example, chemical intuition suggests that, at least to a first approximation, the accessible substrate scope for reduction by a solvated electron should be independent of the **PC** ^•−^ and excitation wavelength used to generate it (Scheme 23b). Even if a solvated electron is initially generated in a ‘hot’ state (which may have a greater reduction potential), subsequent vibrational/translational relaxation should be extremely rapid, and faster than any diffusion‐limited reactivity. In practice, however, the effective scope of photoexcited radical redox reactivity often varies substantially between different **PC** ^•−^ and can depend heavily on excitation wavelength for each given **PC** ^•−^. Notably, this latter dependence tends to be substantially less clear‐cut than photoreactivity simply being consistently ‘off’ upon excitation above a certain threshold wavelength and ‘on’ below it (for example, *cf*. Table 2 in Section 4.1, where reactivity does ‘turn off’ at longer wavelengths, but only for *certain* substrates). It is possible that this more elaborate wavelength‐dependent substrate scope may reflect wavelength‐dependent variations in the apparently complex dynamics of initial electron detachment from **PC** ^•−^, of electron solvation, and of e^−^_(solv.)_ re‐combination with **PC** [120]. Alternatively, these observations may suggest that solvated electron pathways are not the only ones available in these cases, and that some of the reactivity observed is due to parallel, competing mechanisms such as preassembly (potentially including higher D*_n_* photoreactivity) or **PC** ^•−^ degradation, especially at longer wavelengths where e^−^_(solv.)_ are less accessible (*cf*. Scheme 22).

## 6 General Discussion

Perhaps the most important general observation to emphasize at the start of this discussion is that there is, at present, no *single* mechanistic proposal that can account for the full set of experimental observations made during the study of photoexcited radical redox reactions to date. For *reductive* processes, probably the closest to a ‘universal’ model that currently exists in the literature is the solvated electron model discussed in Section 5, given the relatively wide variety of **PC** ^•−^ motifs for which it has been proposed, as well as the availability of a diagnostic NIR signature for e^−^_(MeCN)_, combined with this model's ability to directly account for many observed solvent and wavelength dependences (though not yet *all*; *cf*. Section 5.3).

Another potential advantage of solvated electron mechanisms relates to the phenomenon of back‐electron transfer. For *any* mechanism of photoinduced substrate reduction, the productive quantum yield of this reduction will depend not only on the efficiency of the initial SET step, but also on the relative rates of the desired downstream step *vs*. simple, unproductive SET from **sub** ^•−^ back to ground state **PC** (Scheme 24a). For example, during reductive functionalization of aryl halides, ArX^•−^ formation must generally be followed by (irreversible) fragmentation to generate the desired Ar^•^ intermediate (and X^−^; Scheme 24b). However, if BET is very rapid, this is likely to be outcompeted, leading to low (or even negligible) overall Ar^•^ formation. Notably, when SET occurs directly from a **PC** ^•−^ excited state, BET will be accelerated by the fact that **PC** and **sub** ^•−^ must unavoidably be formed in direct proximity, and BET within the initial solvent cage is therefore not diffusion limited (Scheme 24c; note that this holds true regardless of whether the initial SET occurs via dynamic or static quenching, and identical logic applies to photoactive **PC** ^•−^ decomposition products). In contrast, in the solvated electron model, substrate reduction occurs at an inherently greater spatial separation from the **PC** motif, which should decrease the impact of BET (Scheme 24d; in essence, in order to persist long enough to encounter the substrate, the solvated electron must have *already* avoided immediate recombination with **PC**) [121].

**SCHEME 24:**
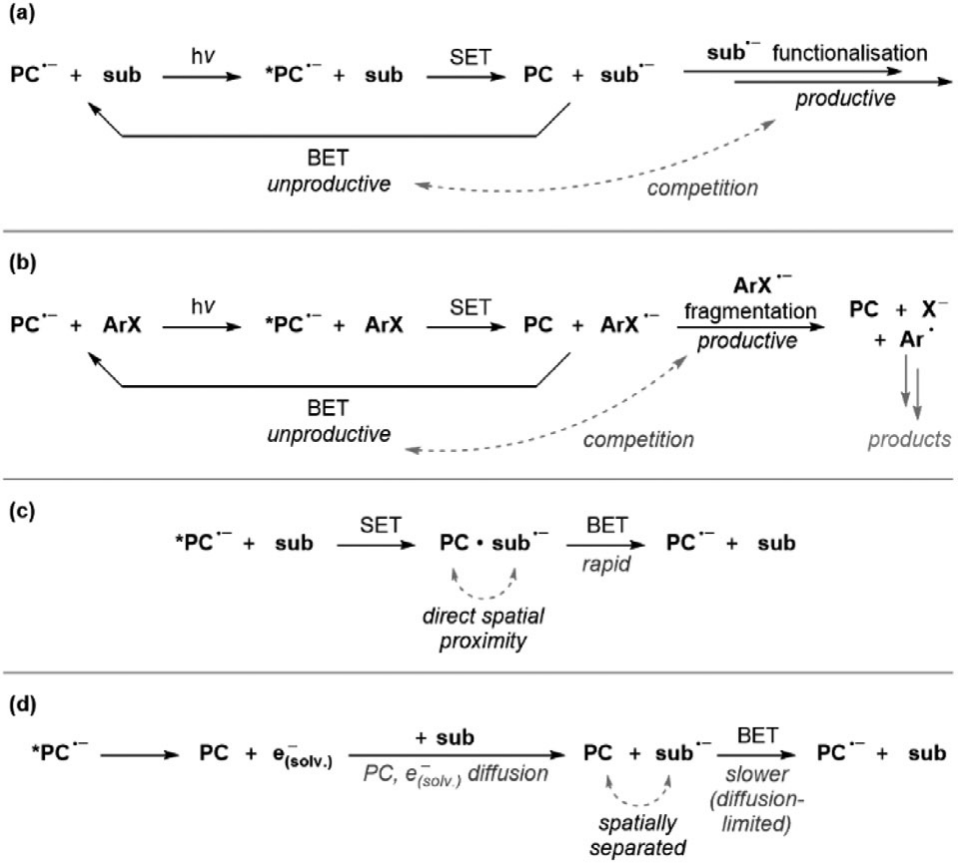
(a) Competition between productive reactivity and unproductive BET following initial reduction of a substrate; (b) aryl halide functionalization as a specific example; (c) acceleration of BET by spatial proximity in most mechanisms; and (d) the absence of such acceleration in solvated electron mechanisms. For simplicity, only the **PC** ^•−^ cases are shown.

Nevertheless, there are a number of stark experimental observations that solvated electron models alone have yet to account for. Perhaps the most obvious of these are reports of (apparent) photoexcited radical redox reactivity under conditions where e^−^_(solv.)_ formation is not expected to be accessible. For example, the Nocera group have reported that e^−^_(MeCN)_ formation from **DCA^•−^** or **NpMI^•−^** requires minimum photon energies corresponding to 400 or 430 nm, respectively [33], and yet reactivity studies with the isolated radical anions have shown clear photoreactivity at wavelengths as long as 730 nm [74]. Meanwhile, our studies of isolated **Acr^•^** have shown evidence for e^−^_(MeCN)_ formation under 395 nm irradiation but not 530 nm, despite photoreactivity being achieved for both wavelengths (*cf*. Table 2 and Scheme 22) [58].

Similarly, while the direct spectroscopic evidence for solvated electron formation is strong in MeCN, there is presently little evidence for equivalent e^−^_(solv.)_ formation in other synthetically relevant organic solvents. This includes solvents such as THF in which photoexcited radical redox reactions can appear to function (albeit often more slowly), but which current understanding suggests are not suitable for solvated electron formation. Equally, there is currently no experimental evidence that any sort of “solvated electron‐like” equivalent model can be applied to examples of *oxidative* photoexcited radical redox [122].

Other experimental observations not currently accounted for using exclusively solvated electron based models include the various ultrafast TAS responses that seem to occur too quickly to be diffusion limited (section 4.2.2), as well as certain structure‐activity relationships that seem to imply an intimate interaction between *PC ^•−^/*PC ^•+^ and substrate (Section 4.2.1). Indeed, the fact that photoexcited radical redox often achieves *selective* transformations (e.g. reduction of an aryl chloride without concomitant Birch‐type reduction of the arene) is conspicuous, given the promiscuous reactivity that might intuitively be expected from a reductant as strong as e^−^_(solv.)_. It is possible that such selectivity could be the result of differences in behaviour occurring *after* the initial SET step (e.g. different relative rates of productive **sub** ^•−^ fragmentation/functionalization vs. unproductive BET; the latter potentially subject to the counterintuitive kinetics of the Marcus inverted region, where SET rates become *inversely* correlated with thermodynamic driving force) [123, 124, 125]. However, at present, any such explanations remain speculative, in the absence of dedicated experimental investigations [126].

Similar limitations can be identified for the other major current models of photoexcited radical redox if one tries to invoke them in the *absence* of solvated electrons (not the least of which being the need to explain away observations of the diagnostic e^−^_(MeCN)_ NIR signal). For example, models based purely on **PC** ^•−^/**PC** ^•+^ degradation suffer due to observations of photoreactivity at wavelengths where degradation products should not absorb, and under conditions where they should not form, as discussed in Section 4.1. Arguably the most holistic current model, which allows rationalization of wavelength dependence in both oxidative and reductive photoexcited radical redox, is preassembly, at least in principle. However, in practice, direct evidence for preassembly currently only exists for a fraction of relevant **PC** ^•−^/**PC** ^•+^ and substrate combinations, and this evidence is often heavily dependent on estimates of maximum diffusion rates. In cases where the discrepancy between predicted maximum rates or efficiencies and those observed experimentally is relatively modest, this interpretation may in principle be vulnerable to any assumptions or simplifications used in the diffusion model. Relevant complicating factors could include non‐ideal solvent behavior; deviation from tabulated diffusion constants due to the presence of electrolytes or high substrate concentrations; differences in **PC** ^•−^/**PC** ^•+^ and substrate diffusion cross‐sections; and any temperature increases caused by the light source, depending on the specific context. One question of particular interest, that so far has not been directly addressed in the photoexcited radical redox literature, is the extent to which these models of diffusion can be applied to solvated electrons. If e^−^_(solv.)_ possess significantly higher diffusivity than more localized **PC** ^•−^ states then the maximum rate for interaction with the substrate would be significantly increased [127]. However, some previous theoretical studies have suggested that this may not necessarily be the case, as e^−^_(solv.)_ diffusion remains limited by the need for solvent reorganization [128]. (In either case, because this is an interaction between e^−^_(solv.)_ and substrate that no longer directly involves the **PC** motif, it would not affect the behavior of the **PC**/**PC** ^•−^ motif itself on faster‐than‐diffusion timescales.)

### 6.1 Competing Pathways

As the discussion above indicates, the current evidence suggests that there may not in fact be a single, common mechanism that is solely operative for all examples of (apparent) photoexcited radical redox. Instead, mechanisms seem to vary on a case‐by‐case basis, depending on the specific **PC** ^•−^/**PC** ^•+^ motif and other synthetic conditions employed. This is interesting, as the most influential studies of photoexcited radical redox mechanisms to date have each tended to focus exclusively on a single mechanistic possibility, with the aim of establishing, in principle, that mechanism's feasibility. Often implicit in this approach has been the expectation—and hope—of finding a single explanation for photoexcited radical redox that can be applied universally. However, as recent developments suggest, the existence of one pathway need not preclude the simultaneous existence of other mechanisms. For example, observation of a solvated electron pathway does not automatically invalidate parallel preassembly or **PC** ^deg^ mechanisms (*cf*. Scheme 22).

Conversely, just because evidence exists to support the feasibility of a particular mechanism for a particular combination of **PC** ^•−^/**PC** ^•+^ and substrate, it does not automatically follow that the same mechanism will be feasible for a different **PC** ^•−^/**PC** ^•+^, or a different substrate. For example, **PC** ^•−^/**PC** ^•+^ degradation pathways may operate successfully but only be able to reduce easier substrates (as discussed for **NpMI•H^−^** and **CA^−^** in sections 3.1 and 4.1). These degradation pathways will also be inherently **PC**‐dependent (e.g. **DCA^•−^** is unstable to Ph^•^, but **NpMI^•−^** reportedly far less so [74]; *cf*. Scheme 10b). Similarly, mechanisms operative at one excitation wavelength may not be accessible at others, even if apparent photoexcited radical redox reactions are observed at both. For example, solvated electron pathways may primarily operate at short wavelengths, while observations that *some* substrates are also reduced at longer wavelengths may be the result of secondary pathways such as preassembly. In other words, competing mechanisms may simultaneously be operative even for a specific combination of substrate and **PC** ^•−^/**PC** ^•+^, with the dominant contributor potentially depending on the choice of solvent, excitation wavelength, and so on (*cf*. Scheme 22). Indeed, Pfund and Wenger have speculated that reaction pathways may vary even during the course of a single reaction [28]. For example, it is easy to imagine a scenario where reactivity initially proceeds via *PC ^•−^/*PC ^•+^, but over time the **PC** motif degrades, and reactivity begins to occur primarily though these degradation products (Scheme 25a) [129]. Similarly, preassembly‐based mechanisms could be favored at early reaction timepoints but diminish in importance later on when the remaining substrate concentration is lower, and preorganized **PC** ^•−^•**sub**/**PC** ^•+^•**sub** complexes are a correspondingly smaller part of the overall **PC** speciation (Scheme 25b). One can also imagine scenarios where mechanistic rationales that until now have been considered separately could be integrated more directly. For example, if some **PC** ^•−^ degradation pathways are proposed to generate new photoreductants of similar strength to the original **PC** ^•−^, and **PC** ^•−^ can react via release of solvated electrons, it stands to reason that the same could be true of these new in situ photoreductants, as well (Scheme 25c).

**SCHEME 25:**
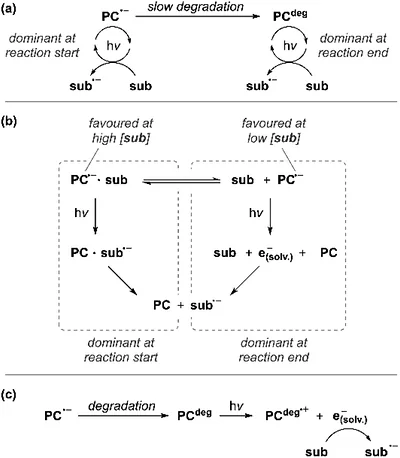
Three plausible mechanistic scenarios that incorporate elements from more than one proposed pathway: (a) transition over time from substrate reduction by **PC** ^•−^ to reduction by a degradation product, **PC** ^deg^; (b) transition over time from static quenching to other mechanisms (e.g. solvated electrons); and (c) solvated electron formation via excitation of **PC** ^deg^.

These considerations lead to the conclusion that future studies of photoexcited radical redox may need to take an increasingly holistic view and consider not just whether a single mechanistic pathway can be operating *in theory*, but rather which out of a range of possible mechanisms are actually occurring *in practice*, and in what proportion.

### 6.2 Are Other Mechanisms Also Possible?

The conclusion that multiple mechanistic pathways may feasibly be available to account for different examples of (apparent) photoexcited radical redox prompts an obvious question: is the current set of mechanistic proposals ‘complete’, or are there still further, undiscovered, feasible pathways, beyond those already described? It is certainly true that, while there have been many experimental observations that are inconsistent with *individual* proposed mechanisms, it is hard to identify any that cannot be explained by some *combination* of these mechanisms. Even so, we would caution against the assumption that there cannot also be other synthetically relevant pathways left to uncover. For example, in situ degradation of different **PC** ^•−^/**PC** ^•+^ motifs can lead to intermediates that are clearly far more diverse than the handful that have been investigated so far (i.e. **NpMI•H^−^**, **CA^−^**; *cf*. Section 3.2). While many of these pathways are likely to be unproductive, it cannot be assumed that this will automatically hold true in all cases [130].

Even without degradation of the **PC** structural backbone, other relevant changes in **PC** ^•−^/**PC** ^•+^ speciation may also still be available. Examples of such structures which to our knowledge have not been considered previously, but for which a mechanistic impact is intuitively plausible, include **PC**•**PC** ^•−^/**PC**•**PC** ^•+^ aggregates (e.g. EDA‐type interactions between electron‐rich **PC** ^•−^ and electron‐deficient **PC** molecules), closed‐shell dimers of **PC** ^•−^/**PC** ^•+^ radicals (i.e. (**PC**)_2_^2−^/(**PC**)_2_^2+^) [131], and closely interacting ion pairs (i.e. M^+^•**PC** ^•−^ or **PC** ^•+^•X^−^; *cf*. previous studies using isolated **PC** ^•−^ which have typically deliberately used counter‐cations designed to be weakly interacting) [74]. The logical extreme of the last of these scenarios can be seen in the context of deazaflavin **PC**s, **dFl**, where conPET has been proposed to occur via photoinduced SET not from **dFl^•−^** but rather the protonated form, **dFl•H^•^**, with this protonation presumably having a significant impact on the radical's stability, redox, and photophysical properties (though to our knowledge there is no indication of a substantial increase in excited state lifetime) [132]. Protonation is also known to facilitate disproportionation of **dFl•H^•^** into **dFl** and doubly‐reduced **dFl•H_2_** (Scheme 26a). Interestingly, Wenger and Li have reported double‐reduction of several **PDI** derivatives, **^R^PDI**, as a strategy to generate very strong closed‐shell photoreductants, **^R^PDI^2−^**, with relatively long, diffusion‐compatible excited state lifetimes of ca. 5–7 ns (Scheme 26b) [133, 134, 135]. While to our knowledge **PC**•H_2_ or **PC** ^2−^ states of this type have not been proposed as active intermediates in any cases of apparent photoexcited radical redox, such mechanisms are not implausible (although it remains unproven whether these are capable of reducing the most challenging substrates that have been employed in synthetic conPET/e‐PRC). Indeed, it is conspicuous that such **PC**•H_2_ or **PC** ^2−^ differ from the “Meisenheimer‐type” **PC**•H^−^ states originally proposed by the Nocera group solely by removal or addition, respectively, of an extra proton (Scheme 26c). Moreover, an analogous disproportionation *has* been proposed as a minor pathway in the oxidation of Cl^−^ by **PTH^•+^** (Scheme 26d) [101]. This disproportionation is proposed to be thermodynamically uphill (limiting its overall mechanistic contribution relative to the dominant **PTH^•+^**/Cl^−^ preassembly pathway discussed in Section 4.2.2), but facilitated by stabilizing ion pairing with Cl^−^.

**SCHEME 26:**
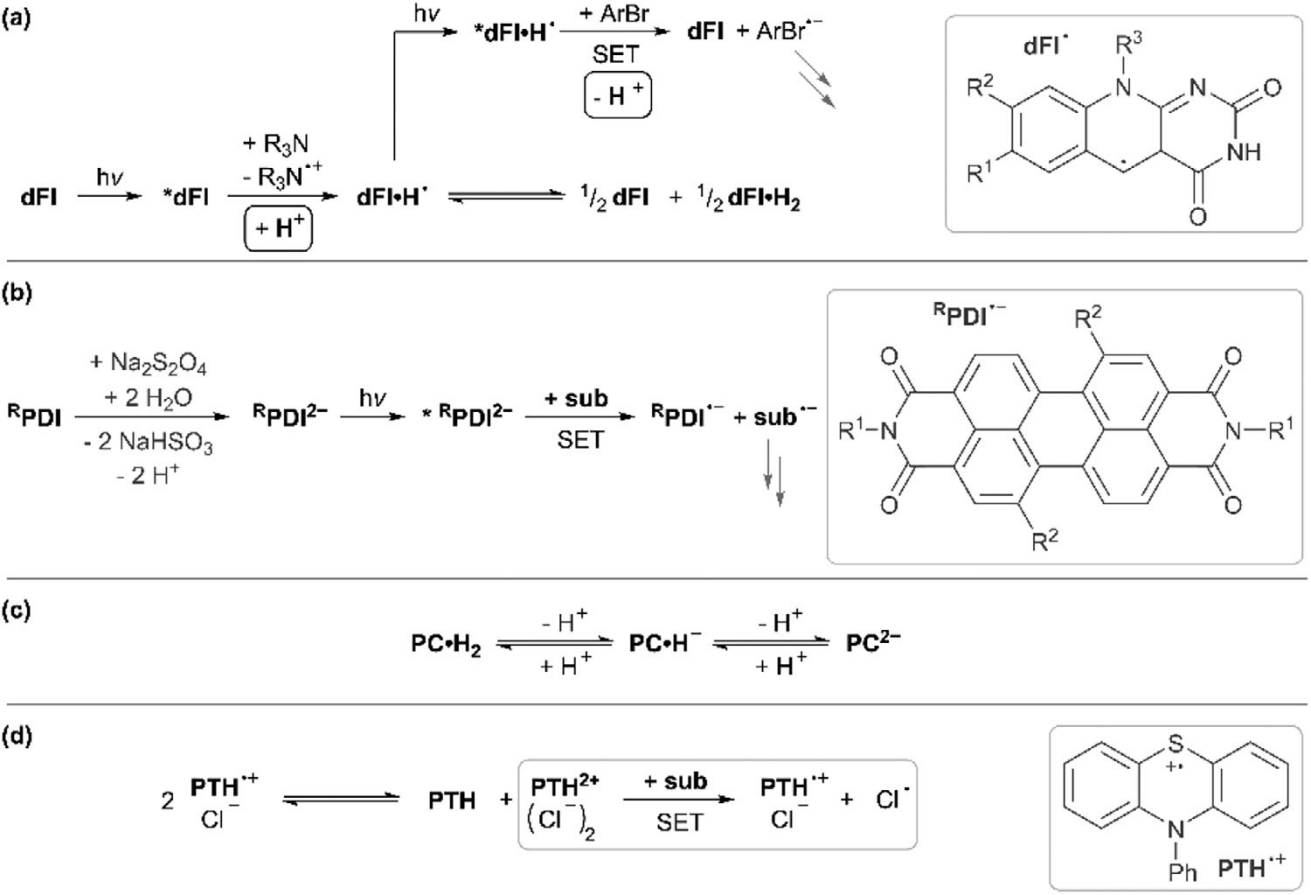
(a) Protonation of a deazaflavin‐derived **PC** ^•−^ state and subsequent photoexcited radical redox reactivity *or* disproportionation; (b) double reduction of **PDI** derivatives to generate strong, closedshell photoreductants; (c) structural analogy between **PC**•H_2_, **PC** ^2−^, and **PC**•H^−^ states, only the last of which have been directly proposed as intermediates in apparent photoexcited radical redox; and (d) Cl^−^ oxidation via disproportionation of a **PC** ^•+^ state.

### 6.3 Synthetic and Mechanistic Consistency

Another conclusion that can be drawn from the observation that photoexcited radical redox mechanisms may vary from case to case is that great care needs to be taken when trying to extrapolate evidence from one system to another. Quite apart from the photoelectronic differences between *different* **PC** ^•−^/**PC** ^•+^, it is increasingly obvious that the photoreactivity of an individual **PC** ^•−^ or **PC** ^•+^ species can be dramatically impacted by even relatively modest changes in reaction conditions. This has been demonstrated clearly for changes in solvent (e.g. Scheme 22), and it seems reasonable to assume that photochemical behavior could be similarly sensitive to related variables such as background ionic strength, counterion identity (for ionic **PC** ^•−^/**PC** ^•+^), and reaction temperature.

As such, wherever possible, future mechanistic investigations should endeavor to study photoexcited radical redox under conditions that directly mimic those employed synthetically. For example, at present, the most detailed time‐resolved spectroscopic analysis in support of preassembly mechanisms has arguably been provided by Pfund and Wenger's studies of **DCB^•−^** and **DCT^•−^** in DMF solution (stabilized by the presence of [*n*Bu_4_N^+^][H_2_PO_4_^−^]; *cf*. Figures 5 and 7 and accompanying discussion) [31, 100]. However, as valuable as these studies are, **DCB** and **DCT** have not been used in any dedicated synthetic reports of conPET or e‐PRC, and it remains an open question to what extent these conclusions can be extrapolated to more synthetically prominent structural analogues such as **DCA^•−^** in MeCN. Similarly, in our own mechanistic studies into the e‐PRC catalyst **NpMI** [74], we have so far largely ignored the possible effects of electrolytes or counter‐cations on the photochemical behaviour of isolated **NpMI^•−^**, despite the fact that this might plausibly have a substantial impact on, for example, e^−^_(MeCN)_ behaviour and BET rates. Meanwhile, essentially all detailed studies of **PC** ^•−^ behaviour have relied on rigorous exclusion of air and moisture to avoid undesired oxidation or other decomposition. Such studies will unavoidably miss any influence caused by trace O_2_ exposure during actual synthetic procedures (*cf*. Section 3.1).

### 6.4 The Spectroscopic Challenge of Studying Photoexcited Radical Redox

Much of the difficulty in understanding photoexcited radical redox can be attributed simply to the challenge of studying these mechanisms spectroscopically. To a certain extent this is true of all photochemical reactions, where insight often unavoidably requires understanding of photoinduced behaviors occurring on timescales far faster than can be monitored by typical, ‘routine’ steady‐state spectroscopic methods such as NMR, ESR, or UV‐vis. However, this is exacerbated for photoexcited radical redox by two major factors. The first of these is the inherent photophysical behavior of the **PC** ^•−^/**PC** ^•+^ states themselves, which unlike closed‐shell **PC** are typically non‐emissive, with extremely short excited state lifetimes (*cf*. Section 2.1). This generally precludes analysis by more routine and widely accessible methods of photophysical characterization such as fluorescence quenching, and forces investigators to instead rely on more specialized and harder to access methods such as ultrafast TAS. This complexity is amplified further by the inherent sensitivity of essentially all relevant **PC** ^•−^ states (though in our experience most **PC** ^•+^ are robust to handling under standard benchtop conditions). This has typically led researchers to rely on in situ methods of **PC** ^•−^ generation, which complicates not only experimental design but also the subsequent spectroscopic analysis and interpretation. This can be avoided by using isolated **PC** ^•−^ prepared ex situ, but doing so introduces the need for air‐sensitive synthesis, sample preparation, and handling. Indeed, spectroscopic studies of both in situ and ex situ generated **PC** ^•−^ usually require careful exclusion of air to prevent undue sample decomposition, complicating experimental design further.

Investigators are challenged again by the fact that many of the proposed mechanisms of photoexcited radical redox also lack diagnostic, easily observable spectroscopic signatures. This has already been discussed for static quenching models (see Section 4.2.3) where, despite being observable in theory, relevant preassemblies are rarely directly observable in practice, forcing assignments to instead rely heavily on inferences drawn from transient spectroscopic observations. Even these TAS signatures seem to vary substantially from case to case (*cf*. Figure 6), with preassembly being proposed to lead to decreased excited state lifetime *or* reduced excited state intensity *or* a persistent **PC** ^•−^/**PC** ^•+^ bleach (with the observed differences possibly being attributed to differences in SET rates, including for BET).

In contrast, solvated electron models *do* offer a specific spectroscopic signature that can be detected, albeit one that occurs primarily in the NIR, outside the typical window of most TAS experiments. It should be noted, however, that solvated electrons are not the only species to absorb in the NIR region; indeed, many relevant *PC ^•−^ states are known to give NIR ESAs (e.g. *4CzIPN^•−^ [45], *DCA^•−^ [33], *Acr^•^ [33]), and this must be factored into assignments of e^−^_(MeCN)_ formation using NIR, including any analysis of e^−^_(MeCN)_ dynamics. As noted by the Ceroni group [45], the dynamic behavior of solvated electrons after generation from **PC** ^•−^ is complex. Understanding the reasons for this complexity, and the corresponding implications for the ability of solvated electrons to engage in productive downstream substrate reduction (potentially even chemoselectively), is currently a major missing piece of this mechanistic model.

Another major blind spot for previous TAS investigations of photoexcited radical redox has been the fate of the substrate. Unlike **PC** ^•−^/**PC** ^•+^, whose backbone structures are designed to give strong UV‐vis responses, the substrates used in synthetic conPET and e‐PRC reactions (and the intermediates and products that derive from them) rarely absorb in this window, which has made them invisible to essentially all previous TAS studies. In the absence of direct observation, substrates’ behavior can instead only be inferred from the behaviour of **PC** ^•−^/**PC** ^•+^ (for example, **PC** ^•−^ oxidation to **PC** may imply substrate reduction to **sub** ^•−^). However, one major limitation of this approach is that it can potentially be difficult to distinguish between productive and unproductive reaction pathways. For example, TAS may show changes in **PC** ^•−^/**PC** ^•+^ behavior that are indicative of an interaction with substrate, without it being clear whether this reaction leads to substrate functionalization of simply regeneration of the **PC** ^•−^/**PC** ^•+^ ground state (*cf*. Scheme 24a). Evidence for unproductive *PC ^•−^/*PC ^•+^ quenching in static quenching studies has already been discussed in Section 4.2.2. However, in principle, this concern can apply equally to solvated electron models. Spectroscopic evidence for substrate reduction in these cases generally relies exclusively on quenching of the NIR signal assigned to the solvated electron. However, without concomitant observation of the substrate this could be consistent with either productive or unproductive pathways. In principle, these two possibilities might be distinguished by consideration of **PC**/**PC** ^•−^ behavior, since a productive pathway should generally [136] lead to persistent **PC** formation (and an equally persistent **PC** ^•−^ signal depletion), while an unproductive pathway should not (and so cause full recovery of the baseline spectrum). However, in general, this has not been explicitly considered in previous reports. Wherever possible, we would therefore encourage future TAS investigations of solvated electron formation to directly report not just NIR behavior attributable to e^−^_(solv.)_ but also UV‐vis behavior attributable to **PC**/**PC** ^•−^. (We note that in the absence of explicit discussion in a report, it is often difficult for interested readers to independently extrapolate from the data as presented.)

Of course, in practice, *PC ^•−^/*PC ^•+^ quenching is unlikely to be either perfectly productive or perfectly unproductive, and TAS observation of apparently completely unproductive quenching may simply reflect interactions where productive quenching does occur, but only as a minor pathway (*cf*. the ca. 10% efficiency estimated for e^−^_(MeCN)_ formation from *4CzIPN^•−^) [45]. Unfortunately, another limitation of many ultrafast TAS experiments is that such minor pathways may be below the effective noise resolution limit, and difficult to deconvolute from the major pathway (especially if signal‐to‐noise is limited by the complexities of in situ **PC** ^•−^/**PC** ^•+^ generation). Indeed, it is notable that for these reasons, despite the prominent role played by quenching efficiency considerations in the initial concerns around photoexcited radical redox feasibility (Section 2.1), spectroscopic studies have struggled to quantify the efficiency of *productive* SET reactions from *PC ^•−^/*PC ^•+^ (as opposed to the overall, ‘productive + unproductive’ quantum efficiency) [137].

#### 6.4.1 Targets for Future Spectroscopic Studies

From the discussion above it is clear that, while understanding of photoexcited radical redox processes has improved dramatically over the past several years, this understanding remains fragmented. Fundamental questions remain to be answered, and there is an ongoing need for further spectroscopic and other mechanistic studies to address these. Of particular value would be any expansion to the range of previously employed experimental methods that would successfully enable:

- direct spectroscopic (or electrochemical) [53, 138] observation of preassembly;
- detailed interrogation of e^−^_(solv.)_ dynamic behavior;
- direct observation of ultrafast substrate behavior (and behavior of substrate‐derived intermediates);
- reliable detection of *minor* *PC ^•−^/*PC ^•+^ quenching pathways; and/or
- quantification of productive vs. unproductive quenching pathways.

### 6.5 Mechanistic Guidelines for Synthetically Focused Studies

The experimental difficulty of studying photoexcited radical redox mechanisms creates a challenge not only for spectroscopists and other mechanistically focused researchers, but also for *synthetic* chemists active in this area. Despite the recent progress made in mechanistic investigations, synthetic applications of conPET and e‐PRC are likely to continue to outpace them, and it is clearly not reasonable to expect each of these synthetic studies to be held up by laborious, comprehensive, hard‐to‐access TAS experiments. Fortunately, preliminary mechanistic information suitable for a synthetically focused study can be obtained much more easily, using relatively simple control reactions. These can provide both meaningful initial insights and a reliable foundation for more detailed subsequent TAS studies if needed. As a robust minimum, we would suggest the following short series of experiments:

- monitoring **PC** ^•−^/**PC** ^•+^ in solution in the dark in the absence of substrate(s) (to establish ground state stability and persistence, especially if generated in situ);
- monitoring **PC** ^•−^/**PC** ^•+^ in solution during or after continuous irradiation in the absence of substrate(s) (to establish photostability);
- monitoring **PC** ^•−^/**PC** ^•+^ in solution in the dark in the *presence* of substrate(s) (to test for ground‐state reactivity); and
- monitoring **PC** ^•−^/**PC** ^•+^ in solution during or after continuous irradiation in the presence of substrate(s) (to test for excited state reactivity).

Ideally, this monitoring should be performed by both NMR and UV‐vis spectroscopy, since both are simple and readily available and they allow complementary monitoring of substrate and **PC** ^•−^/**PC** ^•+^, respectively (with concomitant **PC** formation being detectable by both). Other methods (e.g. ESR) may also be employed if easily accessible. Substrate concentrations should be chosen to match those used synthetically (i.e. in excess relative to **PC** ^•−^/**PC** ^•+^). By default, O_2_ should be excluded as far as possible, especially for reductive transformations, though researchers may wish to repeat reactions under less rigorously air‐free conditions if this is more representative of synthetic procedures, to test the mechanistic impact of adventitious O_2_. These investigations may be performed either using authentic, isolated “ex situ” **PC** ^•−^/**PC** ^•+^ [139, 140], or by generating **PC** ^•−^/**PC** ^•+^ in situ from **PC** by mimicking the redox step used synthetically (e.g. for **PC** ^•−^, electrochemical reduction for an e‐PRC reaction or irradiation in the presence of a weak terminal reductant for conPET). The former, ex situ approach creates an additional synthetic burden, and usually requires access to (and expertise with) dedicated air sensitive synthesis equipment that may not always be available (e.g. gloveboxes). However, it also simplifies experimental design while allowing for highly controlled reactions that are easily interpreted. Conversely, in situ approaches are often more accessible, and may be a better match for the reaction conditions employed synthetically. For example, thin‐film spectroelectrochemistry provides a convenient and flexible way to generate **PC** ^•−^/**PC** ^•+^ with concentrations and optical densities suitable for direct steady‐state and/or transient spectroscopy [97]. However, the more complex mixtures involved mean that these results must be interpreted with slightly more care and caution (for example, in conPET the terminal redox agent may produce non‐innocent byproducts that can interact with **PC** ^•−^/**PC** ^•+^). It is particularly important to confirm that in situ methods lead to *clean* generation of **PC** ^•−^/**PC** ^•+^ from **PC**; i.e. that there are no new minor NMR peaks or UV‐vis maxima that suggest side‐reactivity. Close attention should be paid for any signs of such side‐reactivity during analysis of reactions (i)—(iv) above.

In the case of a genuine photoexcited radical redox reaction, experiments (i)—(iii) should generally lead to no reaction, with reaction (iv) leading to clean consumption of the substrate and **PC** ^•−^/**PC** ^•+^, and formation of **PC** and the final reaction product(s), each of which should be detectable by NMR and/or UV‐vis. In this scenario, assignment of a photoexcited radical redox reaction can be made reasonably confidently, although these experiments cannot inherently distinguish between (for example) dynamic quenching, static quenching, or solvated electron mechanisms (unless a steady‐state spectroscopic perturbation is observed for the **PC** ^•−^/**PC** ^•+^ mixture with substrate that might indicate preassembly). However, if any evidence for decomposition of the **PC** motif is detected then degradation mechanisms may be operative as well (or instead), and so only more tentative conclusions should be drawn.

In cases where it is not already known, investigators may also wish to establish the basic photophysical behavior of the key **PC** ^•−^/**PC** ^•+^ state in question, and especially its excited state lifetime. This may be because the synthetic investigation has explored a new and therefore uncharacterized **PC** ^•−^/**PC** ^•+^ motif, or an established **PC** ^•−^/**PC** ^•+^ whose lifetime has simply not previously been reported. However, such investigations can be laborious in the absence of an emissive excited state. Moreover, we would advise practitioners to be extremely wary of assigning any emissive behavior to a **PC** ^•−^ or **PC** ^•+^ state, especially if this emission is being used as a basis for excited state lifetime estimation or observation of quenching by a substrate. While in principle not impossible, in practice every previous assignment of emission to a relevant **PC** ^•−^/**PC** ^•+^ state that has then been studied in more detail has been proven to be erroneous, due either to more complex photoinduced behavior (at best; *cf*. Scheme 6) [58] or to sample contamination (at worst, and the more frequent scenario; *cf*. Scheme 4) [48]. Any claim of genuine emission from *PC ^•−^/*PC ^•+^ should therefore be considered extraordinary and so demand extraordinary proof.

Finally, in some cases investigators may wish to establish the wavelength dependence of their observed photoreactions, as an indicator for possible e^−^_(solv.)_ or D*_n_* (*n* > 1) involvement. In principle, this can be done simply by switching the LEDs (or other light source used) to ones of a different wavelength and repeating the reaction of interest. However, it is important to note that LEDs of different wavelengths, even ones that at a glance may seem to be of similar power, can provide significantly different photon flux (potentially by orders of magnitude), with blue LEDs typically being more power‐efficient than red. Thus, an *apparent* improvement in performance at shorter wavelengths may simply be due to these LEDs providing far greater photon flux, rather than any true wavelength dependence. Thus, practitioners should ideally measure the relative output of the LEDs in question (e.g. by measuring relative intensity when held at a fixed distance from a detector), and we strongly encourage publication of these results as part of the standard supplementary/supporting information. Outcomes may also be affected by differences in the **PC**/**PC** ^•−^/**PC** ^•+^ absorption profile. If a chromophore absorbs more strongly at a certain wavelength, more efficient light harvesting may lead to greater generation of the excited state, without actually changing this state's identity or behavior. Wavelength dependent reactivity should thus only be assigned if the observed differences in synthetic outcomes clearly outweigh any differences in LED output and/or extinction coefficient. In the clearest cases, this will usually mean reactivity being completely ‘turned off’ at certain wavelengths.

## 7 Summary and Outlook

Although many important details remain unclear, understanding of photoexcited radical redox processes, as well as closely related alternative mechanisms, has evolved dramatically over the past few years, being built up by a series of detailed studies from a wide variety of research groups. Summarizing this combined body of work leads to three overarching conclusions, which can provide guidelines for future investigations in this area:

- Rather than assuming a single operative mechanism is active in all cases, future studies should increasingly consider the potential for multiple pathways to be *simultaneously* accessible and aim to understand which of these are dominant and most productive.
- Results may vary substantially on a case‐by‐case basis, depending on the choice of **PC** ^•−^/**PC** ^•+^, substrate, excitation wavelength, and other reaction conditions. Mechanistic studies should therefore try to *match synthetic conditions* as closely as possible, and great care should be taken when trying to extrapolate from one system to another.
- There remains an acute need for more *detailed spectroscopic studies* [43], both to reinforce and deepen existing understanding and to begin filling in the field's remaining mechanistic blind spots.

We expect that the study of photoexcited radical redox will continue to flourish in the near future, and we hope that our ever‐deepening mechanistic understanding will help to guide and accelerate the continuing expansion of this remarkable field of contemporary synthetic chemistry.

## Author Contributions

**Samuel J. Horsewill**: Writing – review and editing, conceptualization. **Ryan Phelps**: Writing – review and editing, conceptualization. **Joshua P. Barham**: Writing – review and editing, conceptualization. **Daniel J. Scott**: Writing – original draft, conceptualization, writing – review and editing.

## Conflicts of Interest

The authors declare no conflicts of interest.

## Data Availability

Data sharing not applicable to this article as no datasets were generated or analyzed during the current study.
